# Stepwise recruitment of chaperone Hsc70 by DNAJB1 produces ordered arrays primed for bursts of amyloid fibril disassembly

**DOI:** 10.1038/s42003-025-07906-2

**Published:** 2025-03-30

**Authors:** Jim Monistrol, Joseph G. Beton, Erin C. Johnston, Thi Lieu Dang, Bernd Bukau, Helen R. Saibil

**Affiliations:** 1 Institute of Structural and Molecular Biology, Birkbeck University of London, Malet St, London, WC1E 7HX UK; 2 Center for Molecular Biology of Heidelberg University (ZMBH) and German Cancer Research Center (DKFZ), DKFZ-ZMBH Alliance, Heidelberg, Germany; 3 Present Address: Deutsches Elektronen-Synchrotron (DESY), Centre for Structural Systems Biology (CSSB), Hamburg, Germany; 4 Present Address: Leibniz Institute of Virology (LIV) and Universitätsklinikum Hamburg Eppendorf (UKE), Centre for Structural Systems Biology (CSSB), 22607 Hamburg, Germany; 5 Present Address: Department of Chemistry, Kings College London, London, SE1 1DB UK

**Keywords:** Cryoelectron microscopy, Cryoelectron tomography

## Abstract

The Hsp70 chaperone system is capable of disassembling pathological aggregates such as amyloid fibres associated with serious degenerative diseases. Here we examine the role of the J-domain protein co-factor in amyloid disaggregation by the Hsc70 system. We used cryo-EM and tomography to compare the assemblies with wild-type DNAJB1 or inactive mutants. We show that DNAJB1 binds regularly along α-synuclein amyloid fibrils and acts in a 2-step recruitment of Hsc70, releasing DNAJB1 auto-inhibition before activating Hsc70 ATPase. The wild-type DNAJB1:Hsc70:Apg2 complex forms dense arrays of chaperones on the fibrils, with Hsc70 on the outer surface. When the auto-inhibition is removed by mutating DNAJB1 (ΔH5 DNAJB1), Hsc70 is recruited to the fibrils at a similar level, but the ΔH5 DNAJB1:Ηsc70:Apg2 complex is inactive, binds less regularly to the fibrils and lacks the ordered clusters. Therefore, we propose that 2-step activation of DNAJB1 regulates the ordered assembly of Hsc70 on the fibril. The localised, dense packing of chaperones could trigger a cascade of recruitment and activation to give coordinated, sequential binding and disaggregation from an exposed fibril end, as previously observed in AFM videos. This mechanism is likely to be important in maintaining a healthy cellular proteome into old age.

## Abstract (web summary)

Cryo-EM and tomography of the Hsp70 chaperone system disaggregating amyloid fibrils show that the 2 step activation of the class B J-domain protein DNAJB1 is required for the formation of ordered arrays of chaperones that prime fibril disassembly.

## Introduction

Neurodegenerative disorders are characterised by the accumulation of protein aggregates, for example those formed by α-synuclein (αSyn) in Parkinson’s disease (PD), dementia with Lewy Bodies and Multiple System Atrophy^1^. In these disorders, soluble αSyn monomers can self-assemble to generate unstable small species which can quickly react to form large more stable oligomers rich in β-strands and lastly highly ordered amyloid fibrils^2^. Familial forms of PD, mainly caused by single point mutations or by duplication or triplication of the SNCA gene encoding αSyn, suggest a relationship between αSyn aggregation and neuronal death^3–6^. However, as in other protein aggregation diseases, the mechanism of αSyn toxicity remains unknown. Lewy bodies are cytosolic inclusions mainly composed of organelles, lipids and filaments^7^. Fibrillar aggregates are present in post-mortem brains^8^ and both oligomers and fibrils are neurotoxic and can be transmitted between neurons, leading to disease propagation^9–12^. Moreover, amyloid fibrils can promote αSyn aggregation by secondary nucleation^13^.

The 70 kDa heat shock protein (Hsp70) family plays a critical role in the quality control system by regulating protein folding, activity and degradation, by preventing protein aggregation, and by acting in protein disaggregation^14,15^. Hsp70 functions require cycles of association and dissociation with substrate via its ATPase activity. The Hsp70 ATPase cycle is regulated by two cochaperones: a J-domain protein (JDP) which defines the specificity of Hsp70 for the substrate and stimulates Hsp70 ATPase activity, and a nucleotide exchange factor (NEF), acting substoichiometrically, which controls ATPase cycle progression^15^. In metazoa, the combination of Hsc70 (the constitutively expressed human cytosolic Hsp70), DNAJB1 (a class B JDP), and Apg2 (NEF Hsp110) can disassemble αSyn, tau and huntingtin exon 1 amyloid fibrils by depolymerisation and fragmentation^16–18^. It is thought that the dense arrangement of Hsc70 chaperones bound at the fibril surface causes steric clashes between the chaperones and generates entropic pulling forces that destabilise the fibrils^19^. Fibrils are then disassembled unidirectionally by rapid bursts in which protofilaments are unwound and monomers are extracted^20–22^.

There are specific requirements for the cochaperones in amyloid disaggregation. Apg2 more efficiently enhances disaggregation than smaller NEFs such as Bag1^19^; this is attributed to its larger mass. Apg2 also promotes the recruitment of dense clusters of Hsc70 at one end of the fibrils^20^. The specificity of DNAJB1, or at least class B JDPs, is also essential for disaggregation^16^. The interactions between class B JDP and Hsc70 involve a 2-step mechanism^23^. Initially, the C-terminal EEVD motif of Hsc70 binds DNAJB1. This leads to the release of the DNAJB1 auto-inhibitory helix V from the J-domain. The J-domain can then bind to the Hsc70 ATPase domain and stimulate ATP hydrolysis. Although the reason is still unclear, this 2-step mechanism is necessary for fibril disaggregation. In contrast, class A JDPs recruit Hsc70 in a 1-step mechanism that is ineffective in disaggregation of amyloid fibrils^23^.

The coordinated actions of these 3 chaperones can disaggregate amyloid fibrils in vitro. However, structural information about the mechanism is lacking. Here, we use cryo-electron microscopy (cryo-EM) to investigate the structure of DNAJB1 in complex with αSyn amyloid fibrils. This reveals that DNAJB1 binds in a tightly packed array all along the fibrils with a 40 Å repeat. This packing was also seen with a mutant of DNAJB1 lacking the J-domain and G/F linker (ΔJ-DNAJB1), which does not recruit Hsc70. A more targeted mutation replacing the autoinhibitory binding site on DNAJB1, making it functionally like a DNAJA^23^ supports recruitment of Hsc70 to the fibrils but not disaggregation, and does not yield the organised clusters of Hsc70 at fibril ends that appear to be essential for disaggregation.

## Results

### Low-salt conditions reversibly enhance DNAJB1 binding

**Fig. 1:**
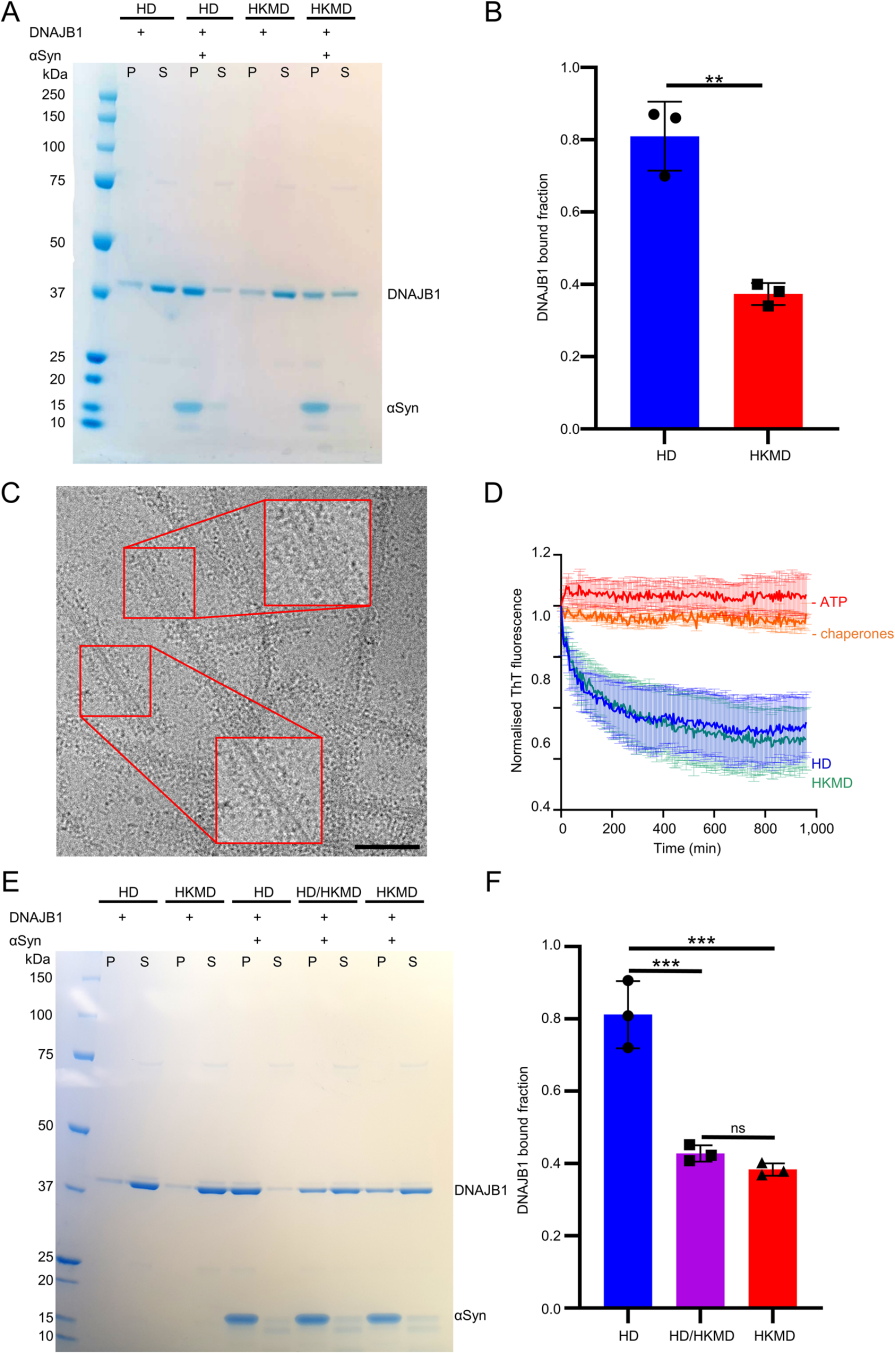
Effect of salt on DNAJB1 binding to αSyn amyloid fibrils. **A** Binding assay for WT DNAJB1 binding to αSyn amyloid fibrils in HKMD or HD buffer. Two controls without amyloid fibrils confirm that salt depletion does not cause precipitation of WT DNAJB1. A larger pellet band for DNAJB1 is observed in HD buffer in the presence of fibrils. S: supernatant, P: pellet. **B** Histogram of WT DNAJB1 bound fractions in each buffer (*N* = 3 independent experiments). A Shapiro–Wilk test was performed to check the normality of the data, followed by an unpaired *t* test (*P* = 0.0016). Mean DNAJB1 binding values with SD are shown. **C** Cryo micrograph of αSyn amyloid fibrils fully decorated by WT DNAJB1 in HD buffer. Scale bar, 500 Å. **D** ThT assay showing that pre-incubation of WT DNAJB1 and αSyn amyloid fibrils in HD buffer does not alter the disaggregation activity. The assay was performed in disaggregation buffer (Table 2). Mean normalised ThT fluorescence is shown with SEM. **E** Binding assay showing the reversibility of the binding when the salts are added back. The proteins were incubated twice in HD buffer (HD condition), twice in HKMD buffer (HKMD condition) or once in HD buffer and once in HKMD buffer (HD/HKMD buffer). **F** Histogram of the WT DNAJB1 bound fraction in each condition shown in (**E**) (*N* = 3 independent experiments). A Shapiro–Wilk test was performed to check the normality of the data, followed by a one-way ANOVA with Tukey’s multiple comparisons test (*P* = 0.0004 between HD and HD/HKMD conditions, *P* = 0.0002 between HD and HKMD conditions). Mean DNAJB1 binding values with SD are shown.

DNAJB1 forms the initial interaction with αSyn amyloid fibrils, binding densely all along the fibrils^16^. However, binding assays of 8 µM DNAJB1 to 20 µM αSyn amyloid fibrils (monomer concentration) in physiological buffer show that only about 40% of the DNAJB1 interacts with the fibrils (Fig. 1A, B, condition in HKMD buffer). The remaining DNAJB1 appears as a high background of unbound protein, hampering image processing of the fibril-DNAJB1 complex. We therefore aimed to optimise DNAJB1 binding to the αSyn amyloid fibrils. DNAJB1 binds to the acidic flexible C-terminus of αSyn and its interactions with the amyloid substrate are mostly electrostatic^19,24^ Therefore, we reasoned that DNAJB1 binding could be increased by depleting salts from the buffer. We investigated the effect of each individual salt and concluded that even 5 mM MgCl_2_ was sufficient to reduce the binding of DNAJB1 (Supplementary Fig. 1A, B). We compared DNAJB1 binding in the high- and low-salt buffers (HKMD and HD, respectively, HKMD: 50 mM HEPES, 50 mM KCl, 5 mM MgCl_2_, 2 mM DTT, pH 7.50; HD: 50 mM HEPES, 2 mM DTT, pH 7.50). In HD buffer, using the same protein concentrations as above, 80% of the DNAJB1 binds to the amyloid fibrils (Fig. 1A, B), doubling the amount of DNAJB1 bound. Cryo-EM of the complex in HD buffer (Fig. 1C) shows a dense fuzzy decoration all along the fibrils and a greatly reduced background, favouring this condition for single particle analysis. We checked that i) this low-salt buffer did not denature DNAJB1 by pre-incubating αSyn amyloid fibrils with DNAJB1 in HD or HKMD buffer before performing a ThT assay in disaggregation buffer (HKMD buffer, 5 mM ATP, 6 mM PEP, 20 ng/µL pyruvate kinase) with Hsc70 and Apg2 (Fig. 1D) and ii) showed that the additional binding is reversed when the salts are added back to the buffer after a first incubation of αSyn amyloid fibrils with DNAJB1 in HD buffer (condition HD/HKMD in Fig. 1E, F). Cryo-EM images of the complex showed reduced contrast and DNAJB1 binding in HKMD compared to HD buffer (Supplementary Fig. 1C, D). Since the HD buffer does not denature DNAJB1 and does not create irreversible interactions, this buffer was used to study the complex formed of wild-type (WT) DNAJB1 and αSyn amyloid fibrils by cryo-EM.

### DNAJB1 binds to αSyn amyloid fibrils in an asymmetric position with a 40 Å repeat

**Fig. 2:**
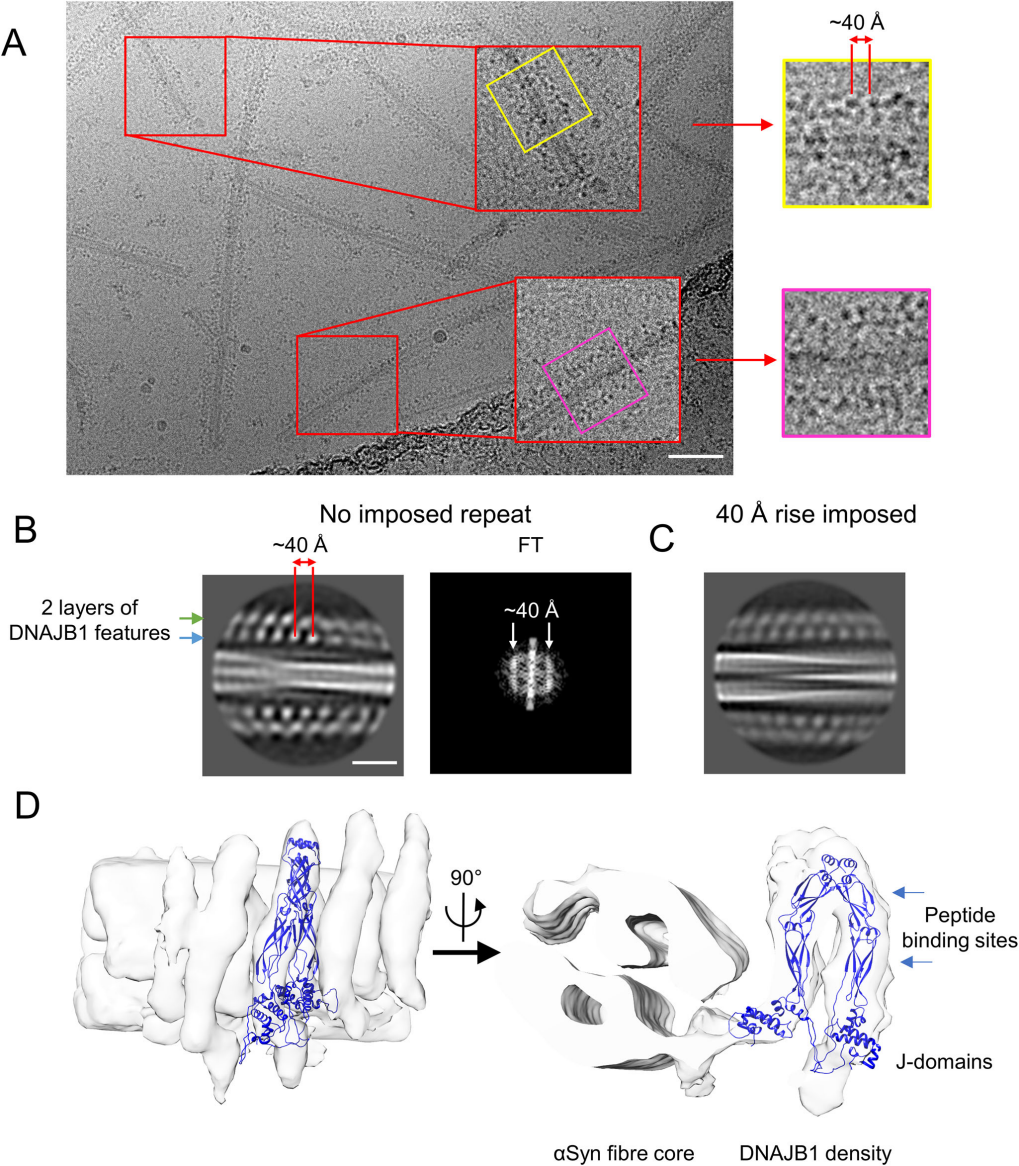
The complex of αSyn amyloid fibrils and WT DNAJB1. **A** Micrograph showing the fuzzy decoration of WT DNAJB1 on αSyn amyloid fibrils. DNAJB1 repeats, displayed as dark dots, are separated by 40 Å. Scale bar, 500 Å. **B** 2D class and FT showing the 40 Å repeat in WT DNAJB1 decoration, shown in reverse contrast. Scale bar, 100 Å. **C** 2D class with a 40 Å repeat imposed for the helical rise. The 2D class is similar to the one displayed in (**B**) with 2 layers of dots characterising WT DNAJB1 decoration on the fibril. **D** Reconstruction of the WT DNAJB1:αSyn fibril complex. The density corresponding to WT DNAJB1 was manually fitted with the crystal structure of the truncated human DNAJB1 lacking the J-domain and G/F linker (PDB: 3AGY) and the NMR structure of DNAJB1 J-domain (PDB: 1HDJ).

We performed cryo-EM single particle analysis to study DNAJB1 binding to the fibrils. The raw micrographs showed the fibrils decorated with two layers of dots, with a ~ 40 Å periodicity along the fibril axis (Fig. 2A). This observation was confirmed on the 2D classes, in which the layered dots were more pronounced and clearly followed the helical twist of the fibrils (Fig. 2B, C). The corresponding diffraction patterns confirmed the 40 Å periodicity (Fig. 2B). The resolution in the 2D class averages was low because the alignment was dominated by the amyloid fibril rather than the less ordered DNAJB1. Nevertheless, we were able to identify two fibril polymorphs within our dataset by combining visual inspection of 2D classes with the initial 3D model generation using the amyloid fibril reconstruction tool (relion_helix_inimodel2d) in RELION 3.1 (Supplementary Fig. 2). We separated the particles for each polymorph and processed them independently. We did not observe any differences in DNAJB1 binding to the various polymorphs in our preparation (Supplementary Fig. 2). Although DNAJB1 density was visible for both polymorphs, the reconstruction of polymorph 1 showed more clearly resolved DNAJB1 density. We generated a structure of this complex at ~14 Å with a mask applied to focus on DNAJB1 decoration only on one side of the fibril (Fig. 2D and Supplementary Fig. 3A). The DNAJB1 density shows a horseshoe shape oriented in an asymmetric position with one subunit closer to the fibril core. Fitting a model into this horseshoe density showed it is a plausible size and shape for DNAJB1 (Fig. 2D) with the J-domains adjacent to the corresponding CTD (the autoinhibited form). The low-resolution density of the DNAJB1 dimer clearly shows that the gross structure is not changed relative to deposited atomic models, which were obtained in salt-containing buffers. A gap is visible between the fibril core and DNAJB1 peptide binding sites which are ~60–90 Å away from the fibril surface. DNAJB1 interacts with the flexible C-terminus of αSyn, in particular residues 123–129^19^. This leaves 30 disordered residues between the fibril core and the DNAJB1 binding site, which are sufficient to bridge the gap to the inner subunit of DNAJB1. Lastly, reprojections of the map were generated and compared with the 2D classes (Supplementary Fig. 3B, C). They show a reasonable resemblance, supporting the validity of the low-resolution map.

We also examined the αSyn amyloid fibrils alone, by re-extracting the particles for each polymorph, without binning, and reprocessing. In this case, polymorph 2 gave clearer results. We classified the particles without imposing a subunit repeat and the corresponding 2D classes and diffraction pattern displayed the cross-β repeat of 4.8 Å typical of amyloid fibrils (Supplementary Fig. 4A, B). We determined a 3D structure of this amyloid fibril at a nominal resolution of 3.6 Å (Supplementary Fig. 4C, D and Supplementary Fig. 5A, B). For polymorph 1, we could observe apparent side chain density in the core of the fibril, but there was no visible cross-beta separation along the fibril axis. Nonetheless, we were able to identify that our reconstruction was similar to the previously reported polymorph 2b structure (PDB: 6SST^25^)(Supplementary Fig. 2A and Supplementary Fig. 5C). The lack of cross-β separation in our reconstruction precluded model refinement, but we were able to rigid-body dock each protofilament (PDB ID: 6SST) with ChimeraX to generate an approximate model (Supplementary Fig. 5C).

### The J-domain is not required for DNAJB1 binding to the fibrils

The asymmetric orientation of the JDP dimer on the fibril surface was unexpected. To see if the linker and J domain play a role in this binding geometry, we examined the complex formed by αSyn amyloid fibrils with a truncated DNAJB1, lacking the G/F linker and J domain (ΔJ-DNAJB1). It has been shown that the DNAJB1 J-domain alone is sufficient to recruit Hsc70 to the fibrils but does not support disaggregation^19^. We first assessed whether HD buffer would also promote ΔJ-DNAJB1 binding (Supplementary Fig. 6). A binding assay showed that 1) HD buffer doubles the amount of ΔJ-DNAJB1 bound to the fibrils and 2) adding back the salts reverses the additional binding (Supplementary Fig. 6A, B). We also confirmed by cryo-EM that this condition promotes the formation of the complex with little ΔJ-DNAJB1 free in solution (Supplementary Fig. 6C). Nevertheless, the complex formed with ΔJ-DNAJB1 is completely inactive in disaggregation (Supplementary Fig. 7), consistent with the earlier finding that the J-domain is essential for recruitment of Hsc70 to αSyn amyloid fibrils^20^. Moreover, the isolated J-domain (including the GF linker) does not bind to the fibres (Supplementary Fig. 8), confirming that the binding is mediated by the C-terminal domains of DNAJB1.

As for WT DNAJB1, we performed single-particle analysis using the amyloid fibril reconstruction toolbox in RELION 3.1 to study ΔJ-DNAJB1 in complex with αSyn amyloid fibrils. The characteristic 4.8 Å amyloid repeat was visible in 2D classes and their corresponding Fast Fourier Transform (FFT) (Supplementary fig. 9A, B). We then generated a structure at 3.2 Å (Supplementary Fig. 9D and Supplementary Fig. 10) with a helical twist of -1.33° and a helical rise of 4.74 Å (Supplementary Fig. 9C). This structure is different from the two forms shown in Supplementary Fig. 2, but similar to a reported structure with an additional density at the N-terminus displaying a hairpin-like shape (Supplementary Fig. 10, PDB: 6OSJ^26^). However, we could not assign the sequence for the additional hairpin because of a gap in the density.

**Fig. 3:**
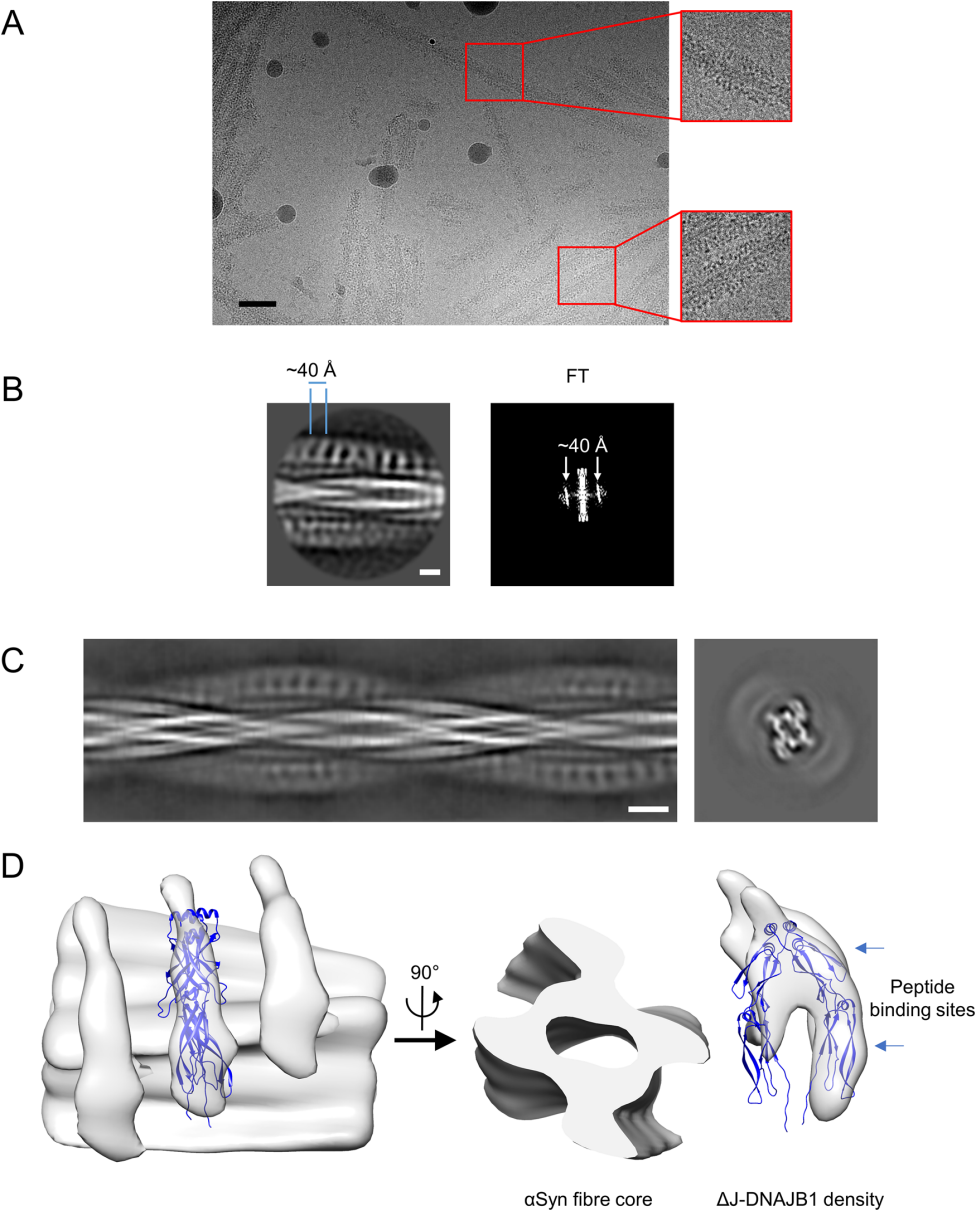
The complex of αSyn amyloid fibrils and ΔJ-DNAJB1. **A** Micrograph showing the fuzzy decoration of ΔJ-DNAJB1 on αSyn amyloid fibrils. ΔJ-DNAJB1 repeats are discernible as dark dots. Scale bar, 50 nm. **B** 2D class and FT showing the 40 Å repeat in ΔJ-DNAJB1 decoration, shown in reversed contrast. Scale bar, 50 Å. **C** Side view of the aligned 2D classes and calculated cross-section. Scale bar, 100 Å. **D** 3D reconstruction of the ΔJ-DNAJB1: αSyn fibril complex. The density corresponding to ΔJ-DNAJB1 was fitted with the crystal structure of the truncated human DNAJB1 lacking the J-domain and G/F linker (PDB: 3agy) using Flex-EM. Scale bar, 50 Å.

We then studied the complex with the ΔJ-DNAJB1 mutant. Like WT DNAJB1, micrographs of ΔJ-DNAJB1 bound to αSyn amyloid fibrils showed a pseudo-regular binding of the chaperone (Fig. 3A). 2D class averages and their power spectra showed the same 40 Å periodicity (Fig. 3B). However, the pattern of decoration seen in projection was different from that of WT DNAJB1: instead of 2 layers of dots, ΔJ-DNAJB1 decoration was characterised by stripes perpendicular to the fibril axis (Fig. 3B,C). Since the globular J-domain and G/F linker represent 46% of DNAJB1 molecular weight (17 kDa of the 37 kDa monomer), the density of the J-domain appears in projection as a bright dot in the 2D classes of the WT DNAJB1. We produced a structure of the complex at ~22 Å, masked to include only one protofilament decorated with ΔJ-DNAJB1 (Fig. 3D and Supplementary Fig. 11A, B). Like the complex with WT DNAJB1, ΔJ-DNAJB1 is positioned asymmetrically relative to the fibril core with one subunit closer to the fibril. The density corresponding to ΔJ-DNAJB1 was fitted with the crystal structure of DNAJB1 lacking the J-domain and the G/F linker, with a similar change in dimer angle to that in full length DNAJB1 (Fig. 3D, PDB: 3AGY). As for the WT DNAJB1 complex, we could not determine a structure at a higher resolution because the fibril core dominated the alignment. Lastly, we checked that reprojections of the map show a reasonable resemblance to the 2D classes, supporting the validity of the low-resolution map (Supplementary Fig. 11B, C).

These structures were obtained using a buffer without salts (HD buffer), to enhance the amount of bound DNAJB1 to the fibrils. Although the HD buffer was not physiological, it did not denature DNAJB1 and the stronger binding was reversed when the salts were added back. Earlier negative stain tomography of the DNAJB1-fibre complex at physiological salt concentration suggested a ~50 Å repeat^16^. The 40 Å repeat of DNAJB1 reported here is based on diffraction peaks from cryo-EM 2D class averages, which are much more accurate than the previous estimate. Therefore, we conclude that the HD buffer did not affect the regularity of the binding but was useful to facilitate single particle analysis.

We attribute the limited resolution of the DNAJB1 maps, at ~14 Å and ~22 Å for WT DNAJB1 and ΔJ-DNAJB1, respectively, to the high flexibility of the 74 kDa DNAJB1 dimer in the complex (around 30 disordered residues separate the rigid core of the fibril from DNAJB1 density) that caused the alignment to be dominated by the highly ordered fibrils. In the case of the ΔJ-DNAJB1 complex, the dimer is only 40 kDa, and the map had to be low pass filtered to retain the weak DNAJB1 density in the 2D class averages.

Despite the low resolution, the similarity in features between the WT DNAJB1 and ΔJ-DNAJB1 complexes, obtained completely independently with different fibril preparations, different polymorphs, different DNAJB1 proteins and by different authors, suggests that the overall features are reliable. The ΔJ-DNAJB1 map shows that the removal of the J-domain and G/F linker does not change the regular, asymmetric binding of DNAJB1 to the fibrils.

### Helix V of the DNAJB1 J-domain is required to produce the organised, active clusters of chaperones at fibril ends

Since DNAJB1 J-domain and autoinhibitory sites are not required for the regular DNAJB1 binding to αSyn amyloid fibrils, we wondered whether the 2-step action of DNAJB1 could influence the pattern of Hsc70 recruitment. It has been shown that only class B JDPs can trigger fibril disaggregation, attributed to the additional auto-inhibitory helix V in the G/F region docking to the J-domain in a 2-step mechanism for Hsc70 recruitment to the substrate^16,23^. Class A JDPs, which do not possess that additional helix, can recruit Hsc70 in a 1-step mechanism but are inactive in disaggregation. We used a mutant of DNAJB1 (ΔH5 DNAJB1), which contains 5 mutations (H99G, M101S, F102G, F105S and F106G) to generate an unstructured G/F region as in DNAJA2, and compared Hsc70 decoration between WT and ΔH5 DNAJB1. All the following experiments were performed in disaggregation buffer containing salts (Table 2) since MgCl_2_ and KCl are required for Hsc70 ATPase activity^27^.

**Fig. 4:**
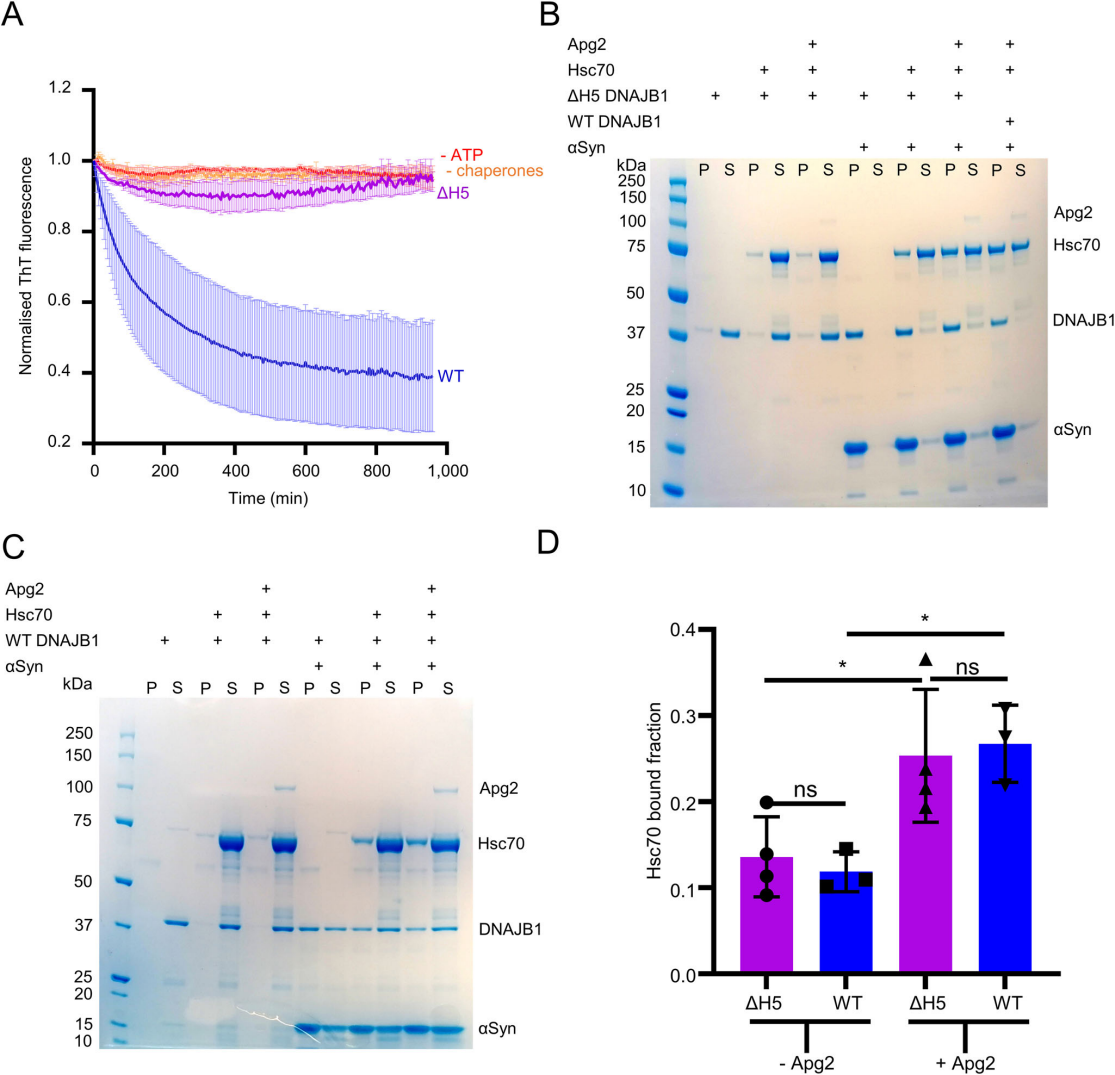
ΔH5 DNAJB1 recruits Hsc70 to the fibrils at the same level as WT but is inactive in disaggregation. **A** ThT assay showing that ΔH5 DNAJB1 cannot trigger the disaggregation of αSyn amyloid unlike WT DNAJB1. The control curves omitting ATP or chaperones are repeated from the ones shown in Supplementary Fig. 7. Mean normalised ThT fluorescence is shown with SEM. **B** Binding assay showing that ΔH5 DNAJB1 can recruit Hsc70 at the same level as WT DNAJB1 in the presence of Apg2. ΔH5 DNAJB1 also enhances Hsc70 recruitment in the presence of Apg2. **C** Binding assay showing that Apg2 can enhance Hsc70 recruitment in the presence of WT DNAJB1. **D** Histogram of the Hsc70 recruitment to the amyloid fibrils as a function of Apg2 and WT or ΔH5 DNAJB1 in each condition shown in (**B**) and (**C**) (*N* = 3 independent experiments). A Shapiro–Wilk test was performed to check the normality of the data, followed by a one-way ANOVA with Tukey’s multiple comparisons test (*P = 0.0142, **P = 0.0014). Mean Hsc70 binding values with SD are shown.

We first checked previously reported results by confirming with a ThT assay that ΔH5 DNAJB1 does not support the disaggregation of αSyn amyloid fibrils, unlike WT DNAJB1 (Fig. 4A). Nonetheless, Hsc70 is recruited to similar levels (~25% and 27%, respectively) in the presence of WT or ΔH5 DNAJB1 and Apg2, as verified by a binding assay (Fig. 4B–D). We noted that Apg2 enhances Hsc70 binding with ΔH5 DNAJB1, as reported for WT DNAJB1^20^ (Fig. 4B, C).

**Fig. 5:**
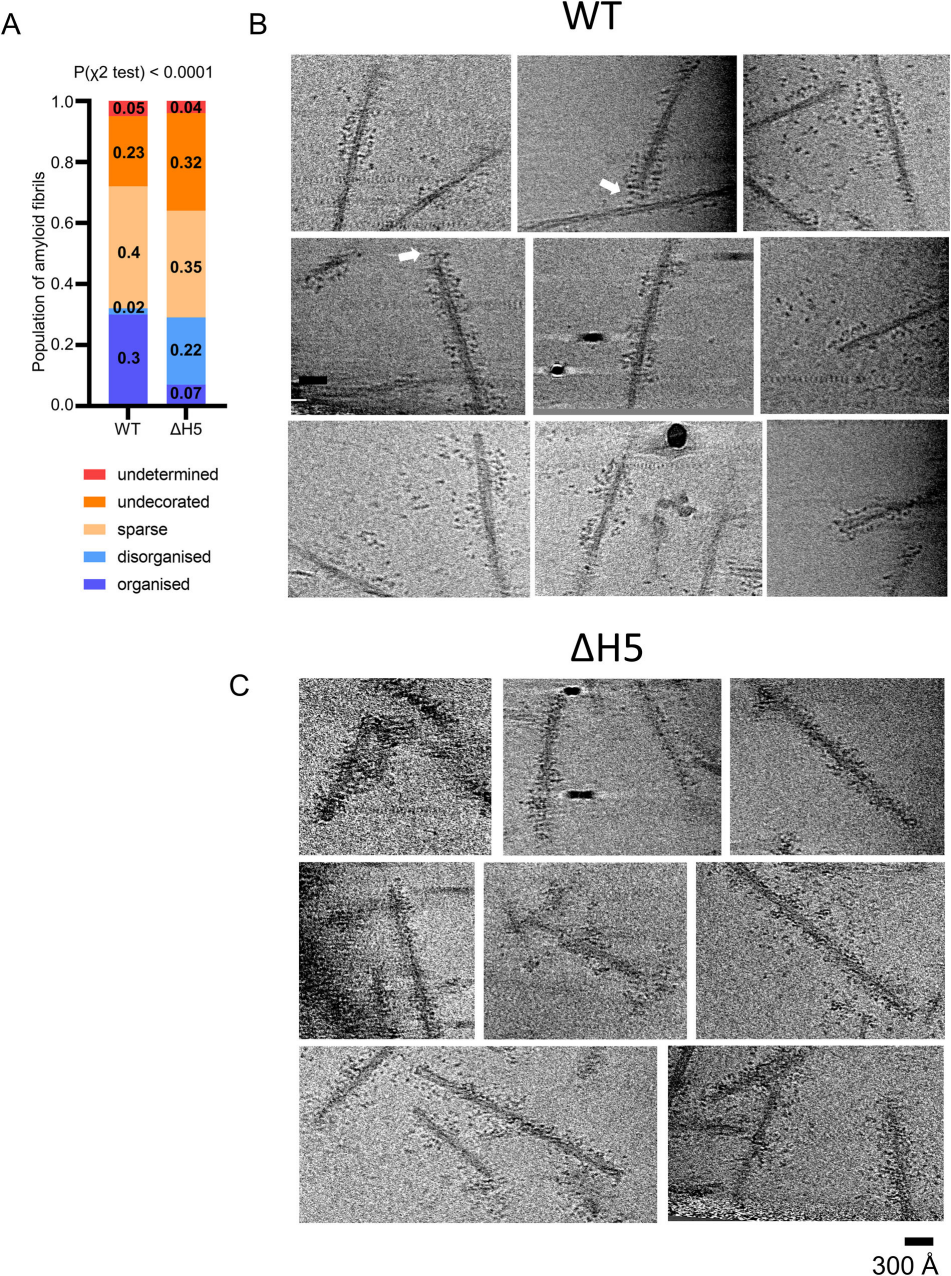
Structural comparison by cryo tomography of WT and ΔH5 DNAJB1 chaperone complexes on αSyn amyloid fibrils. **A** Plot of blinded counting of densely bound regions on the fibril segments, categorised as either organised or disorganised; sparsely bound regions, unbound, or undetermined. The fraction of organised regions is significantly higher in the WT complex (P(χ^2^ test) < 0.0001). Fractional values are shown in the plot and the data are tabulated in Table 1. **B** Tomogram slices of examples of the WT complexes, showing the arrays of chaperones spiralling around the fibrils, with rows of globular densities at the ends of stalks extending from the fibril surface. White arrows indicate examples of the chaperone decoration bending around a fibril end. The maximum diameter of the decorated fibrils is ~390 Å. **C** Tomogram slices of ΔH5 DNAJB1 complexes, showing a less organised binding, with the chaperones more irregularly distributed at different radii. The maximum diameter also reaches 390 Å, but much less consistently than in the WT system. All tomogram slices are averages of 5 sections. Scale bar, 300 Å.

The normal level of Hsc70 recruitment combined with a complete loss of disaggregation activity led us to search for structural differences between the WT and mutant assemblies. The poor signal-to-noise ratio due to the unbound chaperones and the irregularity of the assemblies prevented single-particle analysis (Supplementary Fig. 12) and led us to use cryo-electron tomography (cryo-ET) to compare the structural differences between the two complexes. Complexes composed of fibrils, WT DNAJB1, Hsc70 and Apg2 show highly organised clusters (Fig. 5A, B), whereas the complexes containing ΔH5 DNAJB1 have equally dense, but less regularly distributed decoration on the fibrils (Fig. 5C). The organised clusters contain densely packed globular features tethered to the fibrils, extending radially outwards from the fibril surfaces along spiral tracks following the helical twist of the protofilaments with a spacing of ~50-70 Å. The globular densities extend out to ~140 Å from the fibril surface along the fibrils, estimated by measuring the full outer diameter and assuming a fibril diameter of 100 Å, and often appear lined up in rows. For comparison, DNAJB1 density in the 2D class averages (Fig. 2C; Supplementary Fig. 3) extends ~80-100 Å from the fibril. In the mutant complexes, the recruited densities are scattered over all radii on the fibrils. We quantified the differences in chaperone distribution between WT and ΔH5 complexes by blinded counting (Table 1). The ordered features were seen in 93% (42/45 of the densely bound segments) of the amyloid fibrils densely decorated with WT chaperones, whereas only 25% (23/90 dense segments) of the densely decorated fibrils display organised complexes in the presence of ΔH5 DNAJB1 (Table 1).

**Table 1:** Scoring of fibril decoration

	Organised dense	Disorganised dense	Total dense	Sparse	Not decorated	Not determined	Total
**WT**	30% (42)	2% (3)	32% (45)	40% (55)	23% (31)	5% (7)	138
**ΔH5**	7% (23)	22% (67)	29% (90)	35% (108)	32% (99)	4% (13)	310
**Total**	65	70	135	163	130	20	448

Numbers in brackets and totals are numbers of fibril segments counted. Organised and disorganised categories both show dense binding.

## Discussion

In this study, we have probed the structural basis of amyloid fibril disaggregation by the Hsc70 chaperone system, beginning with single particle cryo-EM to visualise substrate engagement by the DNAJB1 co-chaperone with αSyn amyloid fibrils. The map resolution is very limited by the flexibility of these complexes, in which each component is attached by or near to a long flexible region. Despite this limitation, the maps clearly revealed DNAJB1 binding in an unanticipated, asymmetric position with one subunit of the homodimer closer to the fibril surface, packed with a ~ 40 Å repeat all along the fibrils. This pattern of binding was observed for both WT and ΔJ-DNAJB1 in independent experiments with different fibril conformers. Binding is observed over most of the fibrils, both in the earlier negative stain study^16^ and in the current work by cryo-EM single particle analysis. The binding is consistent with previous findings that i) DNAJB1 binds to the region of residues 123-129 of the disordered C-terminus of αSyn, ii) ΔJ-DNAJB1 can also bind to αSyn fibrils and iii) DNAJB1 binds over most of the fibril length, as previously observed by negative staining tomography^16,19,20^. The approximate fit of DNAJB1 and αSyn (Fig. 2 and Supplementary Fig. 5C) shows that the αSyn C-terminus exits the fibril core at a feasible position for αSyn residues 123 – 129 to contact the DNAJB1 substrate binding domain^19^ (Fig. 2D).

We next considered the role of the two-step auto inhibition of DNAJB1. Since the ΔH5 DNAJB1, mutated to remove autoinhibition by helix 5 in the G/F linker, supported full recruitment of Hsc70 to the fibrils but not disaggregation activity, it was important to compare the structures of the fully assembled, active and inactive complexes on fibrils by cryo-ET. Previous AFM and TIRF studies showed short bursts of disassembly after a period of chaperone recruitment, usually at fibril ends, over a limited length of fibril, 1000–2000 Å^20^. The tomograms show recruited globular densities at some distance from the fibril surface. Although the action of Apg2 clearly increases recruitment of Hsc70 as previously reported (Fig. 4), it is only present at 10% of the level of Hsc70, and is not detected as stably bound on Coomassie stained gels. Therefore we suggest that the recruited globular densities are largely formed of Hsc70. Even if Apg2 were stably bound, Apg2 and Hsc70 are closely related proteins that cannot be discriminated in tomogram slices. In the wild type system the globular densities appear to be held on stalks of density extending away from the fibril, following the helical symmetry of the fibrils (Fig. 5B). On the other hand, with the ΔH5 mutant, the recruited Hsc70 densities are scattered more irregularly over all radii on the fibrils (Fig. 5C). The dense chaperone binding of the full system and disassembly from a fibre end were previously shown by total internal reflection fluorescence (TIRF) and atomic force microscopy (AFM) in our previous study (Beton et al., 2022), as well as by AFM from another group^21^. The cryo-ET results from the current study provide local structural detail rather than the overview and dynamic information of TIRF and AFM. Therefore, we propose that the organised trains of wild type chaperones constitute the hot spots being primed for the bursts of disaggregation previously observed by AFM^20^.

**Fig. 6:**
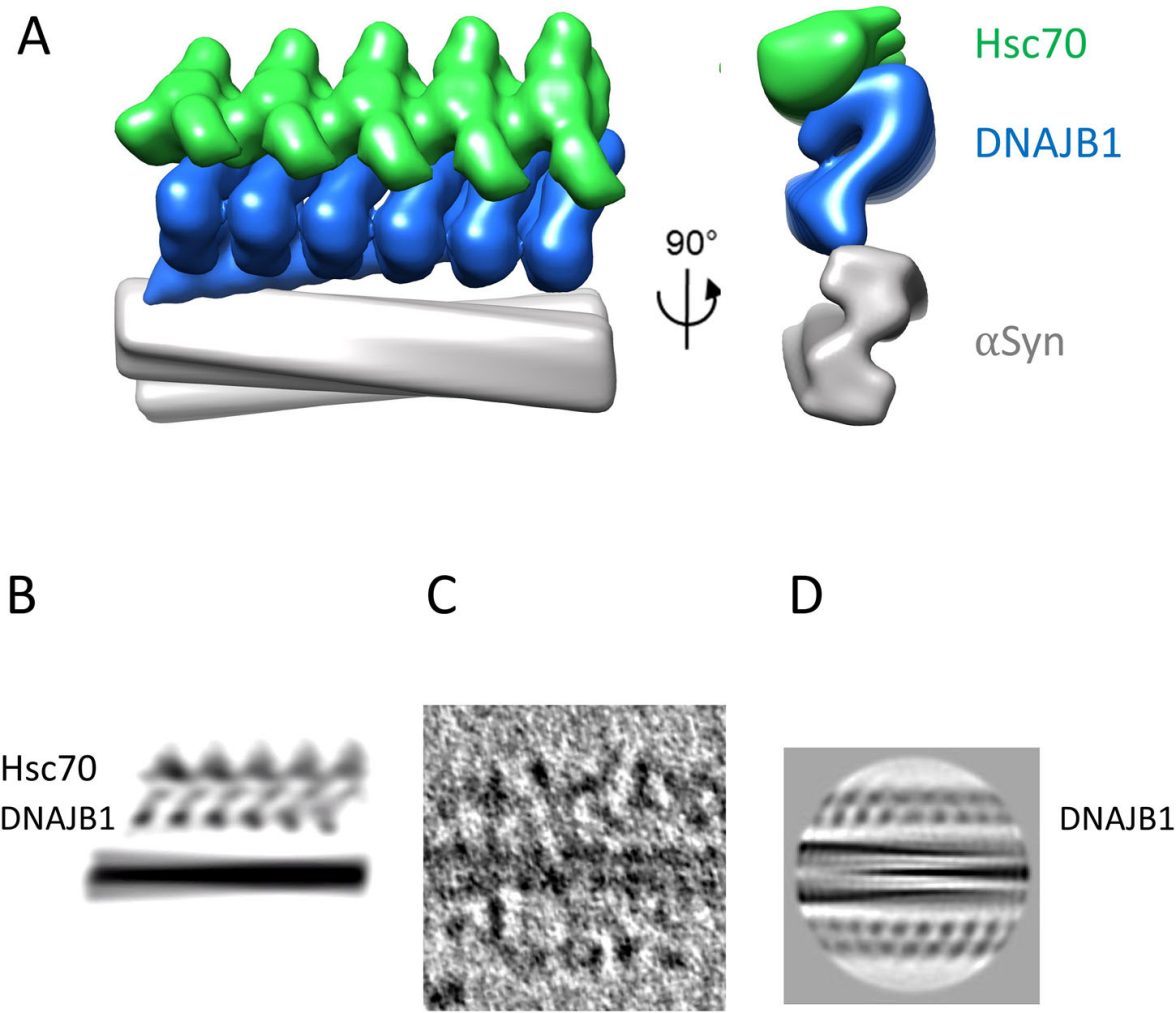
Model of Hsc70 binding and activation. **A** A schematic model of Hsc70 binding to the αSyn:DNAJB1 complex. An alpha fold model of Hsc70:DNAJB1 J-domain was placed on the outside of the complex with the outer J-domain removed from the fitted DNAJB1 model, avoiding clashes. The Hsc70 orientation is arbitrary but it is not possible to place it any closer to the αSyn N termini, because of the presence of the DNAJB1. The model is shown in side and end views. Hsc70 is too bulky to pack every 40 Å as for DNAJB1 and is spaced at 50 Å. The 40 residue long flexible linker to the activated J domain can accommodate a wide range of positions for the Hsc70. **B** Projected density of the model for comparison to the tomogram section in (**C**). **C** Tomogram section of the full chaperone system bound to an αSyn fibril. **D** Class average of αSyn:DNAJB1 reversed in contrast from Fig. 2 (protein density dark) for comparison. The approximate locations of DNAJB1 and Hsc70 layers are indicated with text labels. Panels (**B**–**D**) are shown approximately to the same scale.

In the attempt to build a structural model of the complex, we found that the fibril:DNAJB1 structure places strong steric constraints on Hsc70 binding, despite the uncertainties due to the flexible linkages. The tight packing of the J domains against the C-terminal domains (CTDs) in the DNAJB1:αSyn fibril complex (Fig. 2D) is consistent with the auto inhibited form, and docking of Hsc70 to the accessible J domain via the known Hsp70:J domain interface (eg PDB 5nro) results in severe clashes between the ATPase domain of Hsc70 and the CTDs of DNAJB1 (Supplementary Fig. 13). However, activation of DNAJB1 by binding of the EEVD tail of Hsc70 to the CTD sites releases the J domain^23^, so that it is tethered by a 40 residue flexible linker, allowing it to diffuse up to 120 Å away from the CTD. The Hsc70 can readily bind the released J domain on the outside of the DNAJB1 layer. This constraint allowed us to create a schematic model with the DNAJB1 dimer based on the structure in Fig. 2D, with the outer J domain detached and an alpha fold model of Hsc70:DNAJB1 J domain placed arbitrarily on the outside of the complex, avoiding clashes (Fig. 6A). To assess which features correspond to the DNAJB1 and Hsc70, the density projection of this model (Fig. 6B) is compared to a tomogram section of the full WT complex (Fig. 6C) and to the class average projection of DNAJB1:αSyn fibril (Fig. 6D). Although the precise positions and orientations of the Hsc70 proteins are not known, the tomogram densities and steric constraints of the DNAJB1 clearly locate the bulk of the visible Hsc70 densities over 100 Å from the fibril surface. This leads to the unexpected conclusion that the rows of recruited Hsc70 proteins are too far away to reach their substrate binding sites on the N-termini of αSyn, which are either within 14 residues of the fibril surface (ie a maximum extent of ~40 Å) or on the surface^19^. This leaves the exposed fibril end as the only site at which the Hsc70 can bind its substrate. The tomograms sometimes show the chaperone densities bending around at the fibril ends (white arrows, Fig. 5B). In support of this finding, the previous AFM videos showed zones of dense chaperone binding on the fibrils, which then unravelled from one end after a delay^20^. Since the fibrils are densely covered with DNAJB1 binding sites, the autoinhibition likely coordinates the assembly of the ordered trains of chaperones. These ordered trains could then produce a cascade of disassembly from the exposed fibril end.

**Fig. 7:**
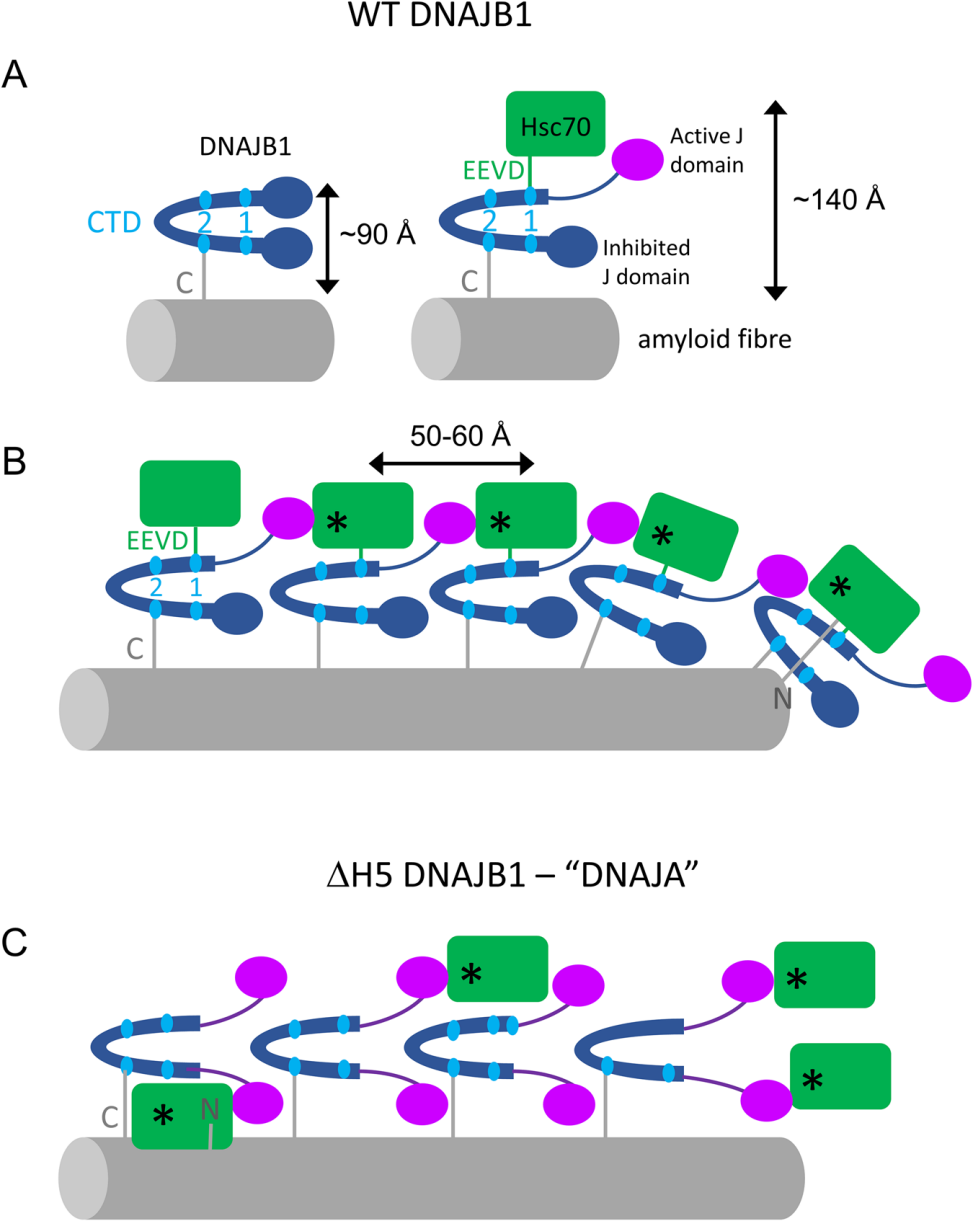
Cartoon showing proposed cooperative, 2-step mechanism of JDP recruitment of Hsc70 into active clusters for disaggregation. **A** WT DNAJB1 binds to the flexible C-terminus of αSyn via its CTD2 site. Then, the EEVD motif of Hsc70 binds CTD1 and releases the J domain, which is proposed to activate an Hsc70 (*) on the adjacent DNAJB1 dimer. This could create a localised cloud of consecutive, actively recycling Hsc70s corresponding to the ordered arrays seen by cryo-ET. **B** The Hsc70s are packed with a spacing of ~50–60 Å along the fibril and extend out >~100 Å from the fibril surface, suggesting that they bind preferentially to the outer subunit of DNAJB1. Only the Hsc70 in complex with DNAJB1 at the fibril end can approach closely enough to interact with the N-terminus of αSyn. The binding follows the helical path of the fibril structure, omitted here for clarity. ATPase cycling is catalysed by Apg2, which is also omitted from the cartoon. It is present at up to 10% of the amount of Hsc70 but is not seen bound to the fibrils in the binding assays. **C** In the ΔH5 mutant system mimicking a class A JDP, all J-domains are already released. Therefore, Hsc70 appears to bind more randomly at all radii, suggesting that it can bind with equal probability to either DNAJB1 subunit. The resulting complexes are not active in disaggregation.

The regions of ordered binding suggest a model, presented as a schematic cartoon in Fig. 7, for the 2-step mechanism in which coordinated binding and activation of Hsc70s are favoured by the close proximity of DNAJB1 dimers. The visible blobs on stalks arrangement is consistent with binding of the Hsc70 EEVD motif to the C-terminal domain I (CTD-I)^19^ of the more accessible, outer DNAJB1 subunit, releasing the auto-inhibitory helix V from the J-domain. This releases the J-domain to activate an Hsc70 by binding its ATPase domain^23^. To form the ordered regions, we speculate that the freed J-domain activates a second Hsc70 bound to an adjacent DNAJB1, which in turn could activate the next Hsc70, as suggested in Fig. 7. The J-domain binding site of DNAJB1 and the C-terminal EEVD site of Hsc70 (which is at the end of a 30-residue disordered tail) can easily bridge between the corresponding binding sites on adjacent DNAJB1 dimers. In contrast, the ΔH5 mutant, acting like a class A JDP, allows uncoordinated binding to all sites and does not produce any disassembly.

The 2-step mechanism could thus trigger a cascade of Hsc70 recruitment and activation, leading to the coordinated formation of organised, dense clusters of chaperones, which, along with ATPase cycling stimulated by transient binding of Apg2, are required for the disaggregation of amyloid fibrils^19,20^.

Considering the possible medical significance of these findings, we can compare our fibril structures to those seen in ex vivo fibrils from patients. In our study, the amyloid fibril cores included residues up to 96 or 97 for the complex with the WT DNAJB1 (Supplementary Fig. 5). The ex vivo structures are ordered until residues 99 and 100 for the Multiple System Atrophy and Lewy Body folds, respectively^8,28^. Since the binding site of DNAJB1 is localised around residues 123-129^19^, this difference does not have an obvious impact on the potential binding of DNAJB1 to patient-derived fibrils. However, the aggregates of αSyn found in Lewy Bodies and Multiple System Atrophy are usually truncated at the C-terminus^29^. The fibrils in such aggregates would likely be deficient in DNAJB1 binding and therefore Hsc70 recruitment, making them resistant to disaggregation by these chaperones^19^.

In summary, we have presented the first structures of a chaperone bound to amyloid fibrils and the first views of the specific effects of class B JDPs at a molecular scale. It remains a future challenge to verify the cooperative formation of organised clusters by class B JDPs and Apg2 and the mechanism of the unidirectional bursts of disaggregation.

## Materials and methods

### Protein purification

αSyn was expressed in DE3 Star *E. coli* cell lines (Thermo Fisher Scientific). Cells were grown in LB media at 37 °C, 180 rpm until reaching an optical density at 600 nm of 0.6–0.8. Protein expression was induced by adding IPTG to a final concentration of 0.5 mM. After induction, cell cultures were incubated for 3–4 h before harvesting the cells by centrifugation (4200 g, 20 min). Cells were lysed by cell disruption in αSyn lysis buffer (Table 2), centrifuged at 48,000 g for 1 h and the soluble fraction was collected. This fraction was then boiled for 20 minutes and centrifuged (21,000 *g*, 30 min). The supernatant was treated with 30 mg/mL streptomycin sulphate, incubated at 4°C for 30 min and centrifuged again (20,000 *g*, 20 min). Finally, the supernatant was treated with 400 mg/mL ammonium sulphate, incubated at 4 °C for 30 min and centrifuged (20,000 *g*, 20 min). The pellet was resuspended in TBS (Table 2) and dialysed overnight into deionised water. The dialysed sample was then purified by ion exchange chromatography with a HiTrap Q HP column using αSyn buffers A and B (Table 2)followed by further size exclusion chromatography (SEC) using αSyn SEC buffer (Table 2) and a Superdex 200 Increase 10/300 column (GE Healthcare). Pure fractions were collected, concentrated up to 1 mM, aliquoted, flash-frozen in liquid nitrogen and stored at -80°C.

**Table 2:** The buffers used in this study

Buffer	Composition
αSyn lysis buffer	100 mM Tris-HCl, 10 mM EDTA, 2 mM DTT, pH 8.0
αSyn buffer A	25 mM Tris-HCl, 2 mM DTT, pH 7.7
αSyn buffer B	25 mM Tris-HCl, 1 M NaCl, 2 mM DTT, pH 7.7
αSyn SEC buffer	50 mM NaPO_4_, 100 mM NaCl, 0.05% w/v NaN_3_, pH 7.30
Hsp lysis buffer	50 mM HEPES, 150 mM KCl, 5 mM MgCl_2_, 2 mM DTT, EDTA free protease inhibitor tablet (Roche), pH 7.50
Hsp wash 1 buffer	50 mM HEPES, 150 mM KCl, 5 mM MgCl_2_, 0.1 mM PMSF, 2 mM DTT, pH 7.50
Hsp wash 2 buffer	50 mM HEPES, 150 mM KCl, 5 mM MgCl_2_, 40 mM imidazole, 2 mM DTT, pH 7.50
ATP wash buffer	50 mM HEPES, 150 mM KCl, 5 mM MgCl_2_, 5 mM ATP, 2 mM DTT, pH 7.50
DNAJ lysis buffer	50 mM HEPES, 750 mM KCl, 5 mM MgCl_2_, 10% glycerol, pH 7.50
DNAJ wash 1 buffer	50 mM HEPES, 150 mM KCl, 5 mM MgCl_2_, 10% glycerol, 40 mM Imidazole, pH 7.50
DNAJ wash 2 buffer	50 mM HEPES, 50 mM KCl, 5 mM MgCl_2_, 10% glycerol, 40 mM, Imidazole, pH 7.50
DNAJ elution buffer	50 mM HEPES, 750 mM KCl, 5 mM MgCl_2,_ 10% glycerol, 500 mM Imidazole, pH 7.50
DNAJ SEC buffer	50 mM HEPES, 750 mM KCl, 5 mM MgCl_2_, 10% glycerol, pH 7.50
HKMD buffer	50 mM HEPES, 50 mM KCl, 5 mM MgCl_2_, 2 mM DTT, pH 7.50
HD buffer	50 mM HEPES, 2 mM DTT, pH 7.50
Disaggregation buffer	HKMD buffer, 5 mM ATP, 6 mM PEP, 20 ng/µL pyruvate kinase

The WT chaperones (DNAJB1, Hsc70, Apg2) were expressed in *E. coli* Rosetta^TM^ DE3 cells with a His_6_-Smt_3_ tag at the N-terminus by identical protocols to those described above for αSyn. Hsc70 and Apg2 were purified by using identical protocols. The cell pellets were resupended in Hsp lysis buffer (Table 2) with an EDTA-free protease inhibitor tablet (Roche). The cells were lysed by cell disruption and centrifuged (48,000 g, 60 min). The supernatant was collected and incubated with Ni-IDA beads for 2 hours at 4°C. Contaminants were removed from the column by performing three washes in Hsp wash 1 buffer, 3 washes in Hsp wash 2 buffer and 2 washes in ATP wash buffer (Table 2). The beads were then incubated overnight at 4°C with Ulp1 protease. The following day, the flow through was collected and the beads were washed twice with Hsp lysis buffer. Flow through and the two washes were pooled and Hsc70 and Apg2 were concentrated up to 100 and 25 µM, respectively. Samples were aliquoted, flash-frozen in liquid nitrogen and stored at -80°C. For DNAJB1, a similar protocol was used, followed by SEC. After binding to the Ni-IDA beads, contaminants were removed using two wash steps: three washes with DNAJ wash 1 buffer (Table 2) and three washes with DNAJ wash 2 buffer (Table 2). DNAJB1 was eluted using DNAJ elution buffer (Table 2) and dialysed overnight in DNAJ lysis buffer with Ulp1 enzyme. The following day, the solution containing cleaved DNAJB1 was reloaded to a Ni-IDA column. The flow through and the first two washes using DNAJ lysis buffer were collected and purified by SEC using DNAJ SEC buffer (Table 2) and a Superdex 200 Increase 10/300 column. Pure fractions containing DNAJB1 were collected, concentrated up to 250 µM, aliquoted, flash-frozen in liquid nitrogen and stored at -80°C.

The purified mutants of DNAJB1 (ΔJ-DNAJB1, the mutant lacking the J-domain and the G/F linker and ΔH5 DNAJB1, mutation of the helix 5 inhibitory binding site: H99G, M101S, F102G, F105S and F106G) were kindly provided by Rina Rosenzweig’s group (Weizmann Institute). Briefly, the mutants were purified by ion metal affinity chromatography (IMAC, Nickel), dialysed and cleaved with TEV protease, subjected to reverse IMAC and then subjected to SEC as for WT DNAJB1. The proteins were stored in 25 mM HEPES pH 7.5, 200 mM KCl, 10 mM MgCl_2_, 2 mM DTT.

### αSyn fibrillation reaction

Monomeric αSyn (200 µM, 1 mL total volume) was incubated in fibrillation buffer (50 mM NaPO_4_, 100 mM NaCl, 0.05% w/v NaN_3_, pH 7.30) in a 1.5 mL Protein LoBind Eppendorf tube (Eppendorf, Germany) on an orbital shaker (PCMT Thermoshaker Grant-bio) at 1,000 rpm, 37°C for 1 week. The formation of amyloid fibrils, rather than amorphous aggregates, was confirmed by negative stain electron microscopy.

### Binding assay

αSyn amyloid fibrils were sonicated for 15 min at high frequency using a CPX 2800 Bransonic Ultrasonic bath (Branson) to maintain them dispersed and chaperone aliquots (WT, ΔJ and ΔH5 DNAJB1) were centrifuged at 17,000 *g* for 30 min at 4 °C to discard any chaperone aggregates. αSyn fibrils (20 µM, monomer concentration) were incubated with DNAJB1 (8 µM) in HD or HKMD buffer (Table 2) for 30 minutes at 30°C. The samples were then centrifuged at 17,000 *g* for 30 minutes. The supernatant was collected and the two fractions (pellet and supernatant) were incubated in 4X NuPAGE LDS Sample buffer (Thermo Fisher Scientific) for at least 30 min at 90 °C. Samples were then loaded on BOLT 4–12% Bis-Tris gels (Thermo Fisher Scientific) and proteins were separated by SDS-PAGE. Densitometry analysis of binding assays was performed using open-source software Fiji. For each biological replicate, band intensities of pellet and supernatant were measured and background was subtracted. The bound fractions were then normalised to the sum of the signals obtained from the pellet and the supernatant for each condition. At least three independent experiments were performed for each condition. The statistical analyses were performed in Prism 8 (GraphPad).

Regarding the reversibility of the binding, a similar protocol was performed with 2 consecutive 30-minute incubations at 30°C in the buffer indicated in the figure. At least three independent experiments were performed for each condition. The statistical analyses were performed in Prism 8 (GraphPad).

Regarding the J-domain with the G/F linker mutant, since the J-domain band and the αSyn band run at very similar heights on the gel, we could not determine the intensity of the J-domain mutant band in the pellet. Therefore, the bound fraction was estimated by determining the fraction of unbound J-domain/total J-domain in the control supernatant without *α*Syn. The bound fraction was then determined as 1-(unbound fraction). At least three independent experiments were performed for each condition. The statistical analyses were performed in Prism 10 (GraphPad).

### Thioflavin T (ThT) assay

αSyn amyloid fibrils were sonicated for 15 min at high frequency using a CPX 2800 Bransonic Ultrasonic bath (Branson). αSyn fibrils (2 µM, monomer concentration) were incubated with DNAJB1 (2 µM, WT or ΔH5 DNAJB1 when indicated), Hsc70 (4 µM), Apg2 (0.4 µM) and ThT (15 µM) in the disaggregation buffer (Table 2). ThT fluorescence was recorded every 5 minutes for 16 h on a FLUOstar Omega plate-reader (BMG LABTECH, excitation: 440 nm, emission: 482 nm). Background ThT fluorescence of chaperones and buffer was subtracted and ThT fluorescence was normalised to the fluorescence intensity of the first time point (*t*  =  0 min). At least three independent experiments were performed for each condition.

Regarding the ThT assay monitoring the effects of the HD buffer on DNAJB1 activity, αSyn amyloid fibrils and DNAJB1 were initially incubated in HKMD or HD buffer for 30 min at 30 °C before performing the ThT assay in the disaggregation buffer in the presence of Hsc70 and Apg2.

### Cryo-EM Sample preparation

The reconstructions of DNAJB1:αSyn presented here were generated from 3 separate datasets. The details for grid preparations in each sample are outlined in Table 3. In all cases, prior to grid preparation, αSyn fibrils (200 μM monomer concentration) were sonicated for 45 min using a Branson 1800 Cleaner or for 15 min at high frequency using a Branson CPX 2800 Ultrasonic bath.

**Table 3:** Details of cryo-EM grid preparation

Datasets	WT DNAJB1 dataset 1	WT DNAJB1 dataset 2	WT DNAJB1 dataset 3	ΔJ-DNAJB1 dataset
**αSyn fibril concentration (μM)**	10	10	6	6
**DNAJB1 concentration (μM)**	20	20	2.4	2.4
**Grid type**	C-flat 2/2	C-flat 2/2	C-flat 2/2, 400 mesh	C-flat 2/2, 400 mesh
**Grid preparation machine**	Vitrobot	Vitrobot	Leica EM GP2	Leica EM GP2

### Cryo-EM Data Collection, refinement and validation statistics

The microscopes and imaging parameters used for collection of each dataset for single particle analysis are summarised in Table 4 and the refinement and validation statistics in Table 5.

**Table 4:** Details of cryo-EM data collection

Datasets	WT DNAJB1 dataset 1	WT DNAJB1 dataset 2	WT DNAJB1 dataset 3	ΔJ-DNAJB1 dataset
**Microscope**	FEI Titan Krios	FEI Titan Krios	FEI Titan Krios	FEI Titan Krios
**Acceleration voltage (kV)**	300	300	300	300
**Camera**	Gatan K2	Gatan K2	Gatan K3	Gatan K3
**Energy filter slit width (eV)**	20	20	20	20
**Super-resolution pixel size (Å)**	0.5025	0.5025	0.55	0.53
**Total frames**	50	50	50	50
**Total dose (e**^**-**^**/Å**^**2**^**)**	44	44	48.5	49
**Defocus range**	1.0 – 3.5	1.0 – 3.5	1.5 – 3.5	1.5 – 3.0
**Number of movies collected**	3261	2797	13429	9,943

**Table 5:** Details of cryo-EM data processing and model refinement

Reconstruction	αSyn fibril from WT DNAJB1 dataset	αSyn fibril from ΔJ-DNAJB1 dataset
Total extracted segments	490,828	911,478
Final extracted segments	245,549	94,945
Helical twist (°)	-0.82	-1.33
Helical rise (Å)	4.76	4.74
Symmetry imposed	C2	C2
Map resolution FSC 0.143 (Å)	3.4	3.2
Refinement		
Initial model used (PDB code)	6RT0	6OSJ
Map sharpening *B* factor (Å^2^)	-105.4	-84.0
Non-hydrogen atoms	4925	5100
Protein residues	735	360
Ligands	0	0
r.m.s.d Bond lengths (Å)	0.006	0.002
r.m.s.d Bond angles (°)	0.833	0.546
MolProbity score	2.05	1.82
Clashscore	9.82	4.53
Rotamer outliers (%)	0.64	0.00
Ramachandran outliers	0.00	0.00
Ramachandran allowed	9.35	11.78
Ramachandran favoured	90.65	88.22

### Helical image processing

All super-resolution movies were gain corrected, binned by a factor of 2, motion corrected and dose weighted using RELION’s CPU implementation of MotionCor2^30,31^. Aligned, non-dose weighted micrographs were used for CTF estimation using CTFFIND4.1^32^. Visual inspection of real-space images and their power spectra was used to identify and discard images showing ice contamination or other defects.

### αSyn fibrils from WT DNAJB1 datasets

Fibrils were manually picked from images from dataset 1 and 2 (Tables 3 and 4) in RELION 3.1^31^ using the helical picking tool and particles were extracted as 320-pixel (336 Å) boxes along the fibril axis with an inter-box distance of 36 pixels (38.4 Å). Reference free 2D classification was used to identify two polymorphs (polymorphs 1 and 2) based on the approximate thickness and helical twist of fibrils in 2D classes. These classes were then used to generate an initial model using the relion_helix_inimodel2d tool^33^, with a helical rise of 4.8 Å for both classes and crossover distances estimated from the raw micrographs. This initial model was lowpass filtered to 10 Å prior to 3D refinements. Reconstructions were generated by multiple rounds of 3D auto-refinement and CTF-refinement in RELION. The final reconstructions were sharpened using RELION postprocessing and the resolution was estimated by Fourier shell correlation (FSC) at 0.143 between the half-maps. The polymorph 2 map was fitted with PDB:6RT0 in Chimera as a rigid body and refined using PHENIX^34,35^.

### αSyn:WT DNAJB1 complex

Particles were picked from images in dataset 3 (Tables 3 and 4) using the filament picking mode in crYOLO 1.5.6. The deep learning network was trained using coordinates manually assigned from 60 micrographs using EMAN2^36^, and this trained network was used to pick particles from the remaining micrographs. 2,300,000 particles were picked using a 420-pixel box size (462 Å) with an inter-box distance of 80 Å. These particles were subjected to multiple rounds of 2D classification and, again, classes corresponding to each αSyn fibril polymorph were separated and processed individually. Initial models were generated using relion_helix_inimodel2d, with the crossover distances used for the fibril only reconstructions. 3D classification was used to identify sets of particles with DNAJB1 density at the fibril edge. Discernible DNAJB1 density was only observed for any classes of the fibril 2 polymorph, and further image processing on the fibril 1 subset was not done. For fibril 1 particles, C2 symmetry expansion was used to increase the effective number of selected particles. These particles were used to generate reconstructions through two rounds of 3D refinement in RELION: firstly, a consensus refinement without applying a mask, followed by a second round of refinement using a mask that incorporated the entire αSyn fibril and the DNAJB1 decoration along only a single protofilament. This reconstruction was sharpened using RELION postprocessing using a user supplied B-factor of -150 Å^2^. A predicted model of the DNAJB1 dimer was generated from the full sequence (taken from UNIPROT entry P25685 using ColabFold^37^. This model was manually placed in density using Chimera^35^ and flexibly refined into the density using Flex-EM^38^. RIBFIND restraints^39^ were generated manually for each domain of DNAJB1 (CTD-I, CTD-II and the J-domain) to prevent overfitting of the model^37,40,41^.

### αSyn fibrils from the ΔJ-DNAJB1 dataset

Particles were picked from images in ΔJ-DNAJB1 dataset (Tables 3 and 4) using the filament picking mode in crYOLO 1.5.6 (Wagner et al., 2019). The deep learning network was trained from 100 micrographs that were manually picked in crYOLO. 911,478 particles were picked using a 420-pixel box size (445 Å) with an inter-box distance of 44.5 Å, binned by a factor 3.3 to yield 128-pixel boxes. These particles were subjected to three rounds of 2D classification. Only particles showing decorated fibrils were selected and the corresponding particles were re-extracted without binning and subjected to two additional reference-free 2D classifications. The classes showing the 4.8 Å cross-β repeat were selected to produce an initial model using the relion_helix_inimodel2d tool, with a helical rise of 4.8 Å and a crossover distance estimated at 620 Å from the raw micrographs. Reconstructions were generated by multiple rounds of 3D auto-refinement and CTF-refinement in RELION. The final reconstruction was sharpened using a B-factor of -84 Å² in RELION postprocessing. The resolution was estimated from FSCs at 0.143 between the half-maps. The map was fitted with PDB: 6OSJ in Chimera as a rigid body, manually refined in COOT and automatically refined in PHENIX^42^.

### αSyn:ΔJ-DNAJB1 complex

The 3.3x-binned particles were subject to 3 consecutive 2D classification without restricting translation along the fibril axis. Best 2D classes were used to generate an initial model using the relion_helix_inimodel2d tool, with a crossover distance estimated at 620 Å. A round of 3D classification was followed by two rounds of 3D auto-refinement. A mask to retain the entire αSyn fibril and only the ΔJ-DNAJB1 decoration along a single protofilament was generated and used for the second 3D refinement. An ad-hoc 25 Å low-pass filter and a B-factor of -196 Å^2^ were applied in the postprocessing stage. The predicted model of DNAJB1 (generated as described above) was truncated (J-domain and G/F linker residues were removed) and manually placed in the density using Chimera. The model was then flexibly refined using Flex-EM with RIBFIND restraints for each domain.

### Cryo-ET sample preparation

Prior to grid preparation αSyn amyloid fibrils were sonicated on high frequency for 30 min using a Branson CPX 2800 ultrasonic bath to produce dispersed fragments of αSyn fibrils. The suspension was diluted to 6 µM αSyn monomer concentration and mixed with 6 µM Hsc70, 3 µM DNAJB1 (ΔH5 or WT) and 0.6 µM Apg2 and incubated for 1 h at 30°C in disaggregation buffer. 4 μL of the wild type preparation were applied to negatively glow discharged holey carbon C-flat grids (CF-1.2/1.3-4 C) (Protochips, USA). 4 μL of the ΔH5 preparation were applied to negatively glow discharged holey carbon C-flat grids (CF-2/2-3Cu-50) (Protochips, USA). Both sets of grids were then back blotted before adding 3 μL of 10 nm Protein-A-coated gold (EMS, USA) as fiducial markers for 3D reconstruction of tilt series. The grids were then back blotted a second time before plunge freezing in liquid ethane using a Leica EM GP2 (Leica Microsystems, Germany).

### Cryo-ET data collection and tomogram reconstruction

The microscopes and imaging parameters used for collection of each dataset for tilt series are summarised in Table 6.

**Table 6:** Details of tilt series data collection

Datasets	WT DNAJB1 dataset 1	WT DNAJB1 dataset 2	ΔH5 DNAJB1
**Microscope**	FEI Titan Krios III	FEI Titan Krios III	FEI Titan Krios III
**Acceleration voltage (kV)**	300	300	300
**Camera**	Selectris Falcon IV	Selectris Falcon IVi	Selectris Falcon IVi
**Energy filter slit width (eV)**	5	3	3
**Pixel size (Å)**	1.9	1.94 (rounded to 1.9)	1.94 (rounded to 1.9)
**Total dose (e/Å**^**2**^**)**	118	116	116
**Defocus range (μm)**	-2 to -6.5	-2 to -4.4	-2 to -4.4
**Number of movies collected**	3261	2797	13429

Tilt series were acquired from +60° to -60° with a 3° increment in a dose symmetric acquisition scheme.

### Reconstruction method

For all tomography datasets, frames underwent whole frame alignment in MotionCor2 version 4.1^30^ and defocus estimation was done using CTFfind version 4.1^32^. Tomograms were not dose weighted or CTF corrected. Tilt series alignment for WT DNAJB1 dataset 1 was performed using Dynamo version 1.1.333^43^. Tilt series alignment for WT DNAJB1 dataset 2 and for ΔH5 DNAJB1 were performed using fiducial based alignment in IMOD version 4.9.0^44^. For all tilt series from WT DNAJB1 dataset 1, motion correction CTF estimation and tilt series alignment were automated using scripts and the guide from the following GitHub repository (https://github.com/EuanPyle/relion4_tomo_robot). For all tilt series alignments from WT DNAJB1 dataset 2 and ΔH5 DNAJB1, motion correction CTF estimation and tilt series alignment were automated using a custom build of the tomography module of Relion 4. All tomograms were binned by 2 in X and Y.

### Counting of fibrils according to type of chaperone decoration

Chaperone binding along the fibrils appeared either dense or sparse, and the densely bound regions appeared either organised, with the bound features spaced at roughly 60 Å, or disorganised, with no discernible regularity or repeat. In order to quantitate these observations, the tomograms of wild type and ΔH5 complexes were randomly mixed and provided without identification for blind counting by one author (Fig. 5). This was done by searching through the planes of each tomogram to find isolated fibrils, not in contact with other structures, that could be scored according to the nature of the decoration.

### Statistical analyses and reproducibility

All statistical analyses were performed using GraphPad Prism 8 or 10 (GraphPad Software). For all the binding assays, at least a biological triplicate was done and a Shapiro-Wilk test was performed to ensure the normality of the data. When the data followed the normal distribution, a one-way ANOVA with Tukey’s multiple comparison test was performed. Otherwise, an unpaired *t* test was performed. Exact *P* values are given in the captions of the figures. Column bar graphs represent the average with the s.d. For the ThT assay, the graphs represent the average with the s.e.m. For the fibril counting (Fig. 5A), a Chi-square test was performed.

### Reporting summary

Further information on research design is available in the Nature Portfolio Reporting Summary linked to this article.

## Supplementary information

Supplementary Information

Reporting Summary

Transparent Peer Review file

## Data Availability

The cryo EM reconstructions and corresponding atomic models are deposited in the EMDB and PDB with the accession codes EMD-19443, 19444, 19448, 19441, 19462, 19461, 19446, 19447 and PDB 8RRR, 8RQM. Raw data for binding, J-domain control and disaggregation assays are in the Birkbeck Research Data Repository at 10.18743/DATA.00000326, 10.18743/DATA.00000349 and 10.18743/DATA.00000328, respectively.
