# Supplementary material for: Stepwise recruitment of chaperone Hsc70 by DNAJB1 produces ordered arrays primed for bursts of amyloid fibril disassembly
Source: Commun Biol. 2025 Mar 30;8:522. doi: 10.1038/s42003-025-07906-2 (PMC11955550; doi:10.1038/s42003-025-07906-2)
Supplement: Supplementary file 1 — Supplementary Information [file 42003_2025_7906_MOESM1_ESM.pdf]

# Supplementary Information

Stepwise recruitment of chaperone Hsc70 by DNAJB1 produces ordered arrays primed for bursts of amyloid fibre disassembly

Monistrol, J, Beton, JB, Johnston, EC, Dang, TL, Bukau, B & Saibil, HR

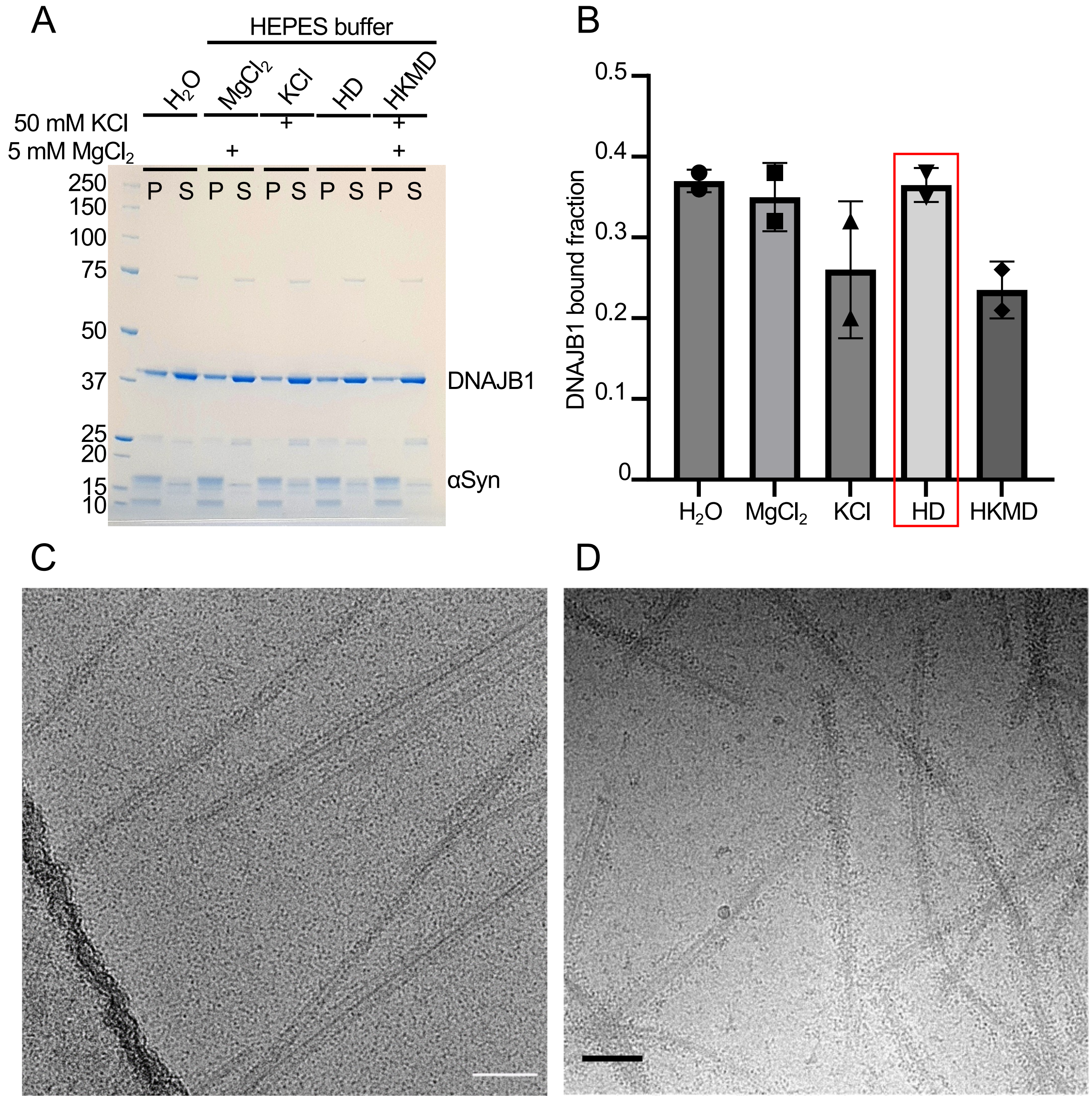


**Supplementary figure 1. Influence of salts on WT DNAJB1 binding to αSyn amyloid fibrils.**

A) Binding assay for WT DNAJB1 to αSyn amyloid fibrils in either deionised water ($H_2O$), 50 mM HEPES + 5 mM $MgCl_2$ + 2 mM DTT pH 7.5 ($MgCl_2$), 50 mM HEPES + 50 mM KCl + 2 mM DTT pH 7.5 (KCl), 50 mM HEPES + 2 mM DTT pH 7.5 only (HD) or HKMD buffer.

B) Histogram of WT DNAJB1 bound fractions in each buffer (N = 2 experiments, average shown with standard deviations). The red frame indicates the conditions selected for the cryo-EM experiments.

C,D) Micrographs of αSyn amyloid fibrils incubated with WT DNAJB1 in either HKMD (C) or HD (D) buffer. Scale bars, 500 Å.

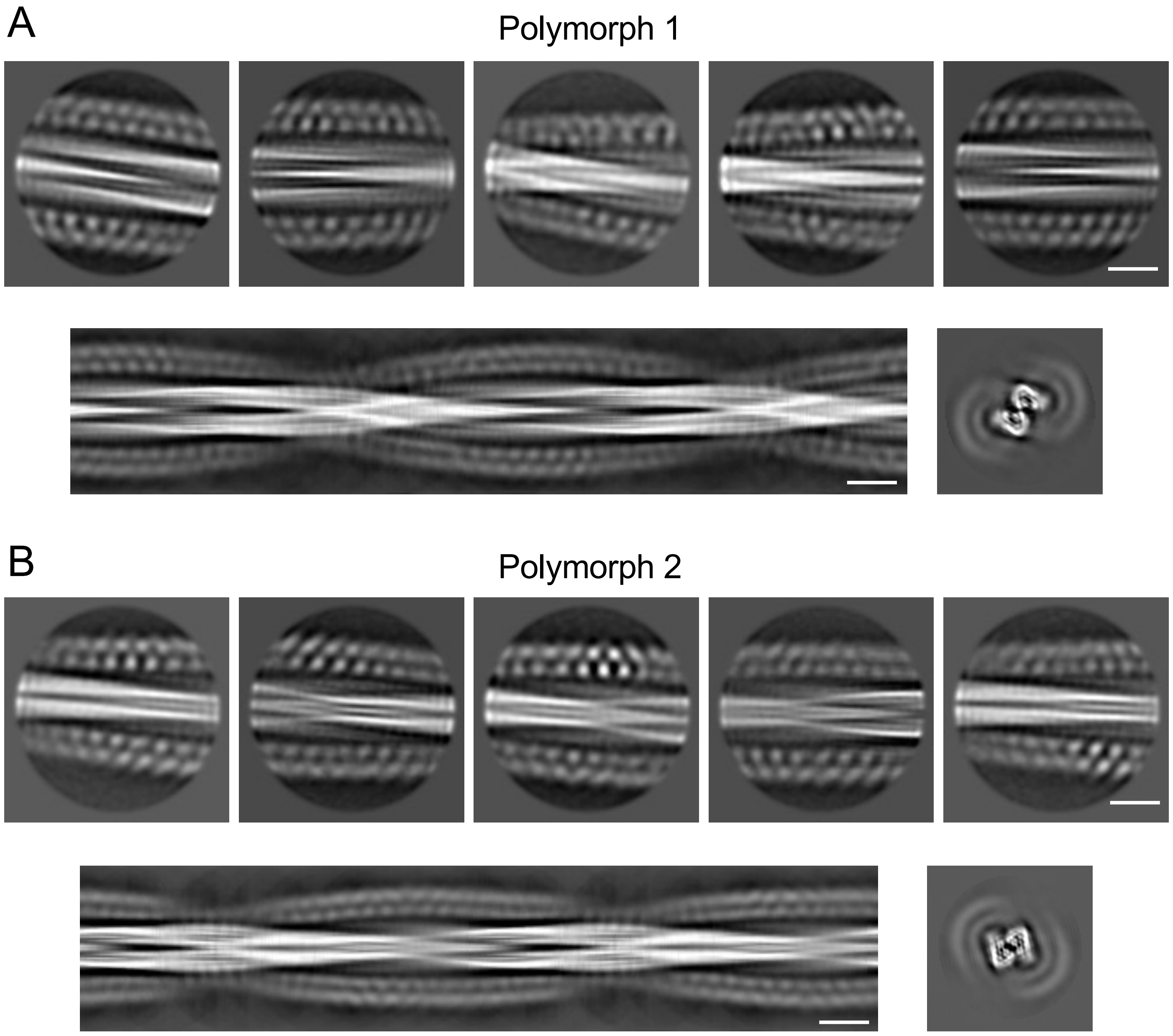


**Supplementary figure 2. Diversity of fibril conformers.**
2D classes, side view of the aligned 2D classes and calculated cross-section for the first (A) and second (B) polymorphs. Scale bars, 100 Å.

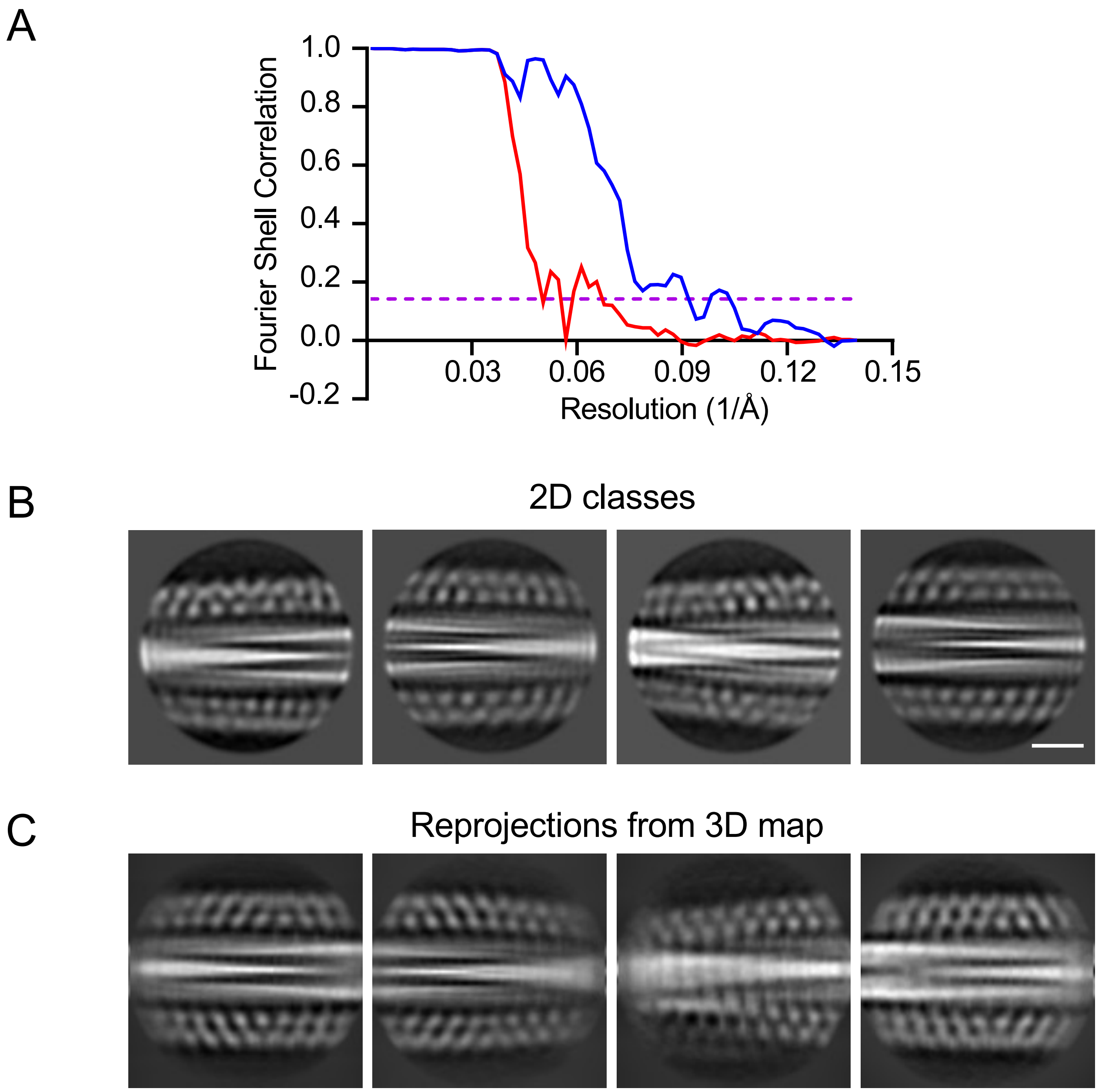


**Supplementary figure 3. Resolution of WT DNAJB1:αSyn fibril complex map, 2D classes and reprojections from the map.**

A) FSC curves of two independently refined half-maps showing phase randomisation (red curve) and the cryo-EM reconstruction (blue curve). The dashed purple line indicates FSC = 0.143

B) Additional 2D classes showing the decorated αSyn fibrils. Scale bar, 100 Å.

C) Reprojections calculated from the 3D map. The reprojections resemble the 2D class averages.

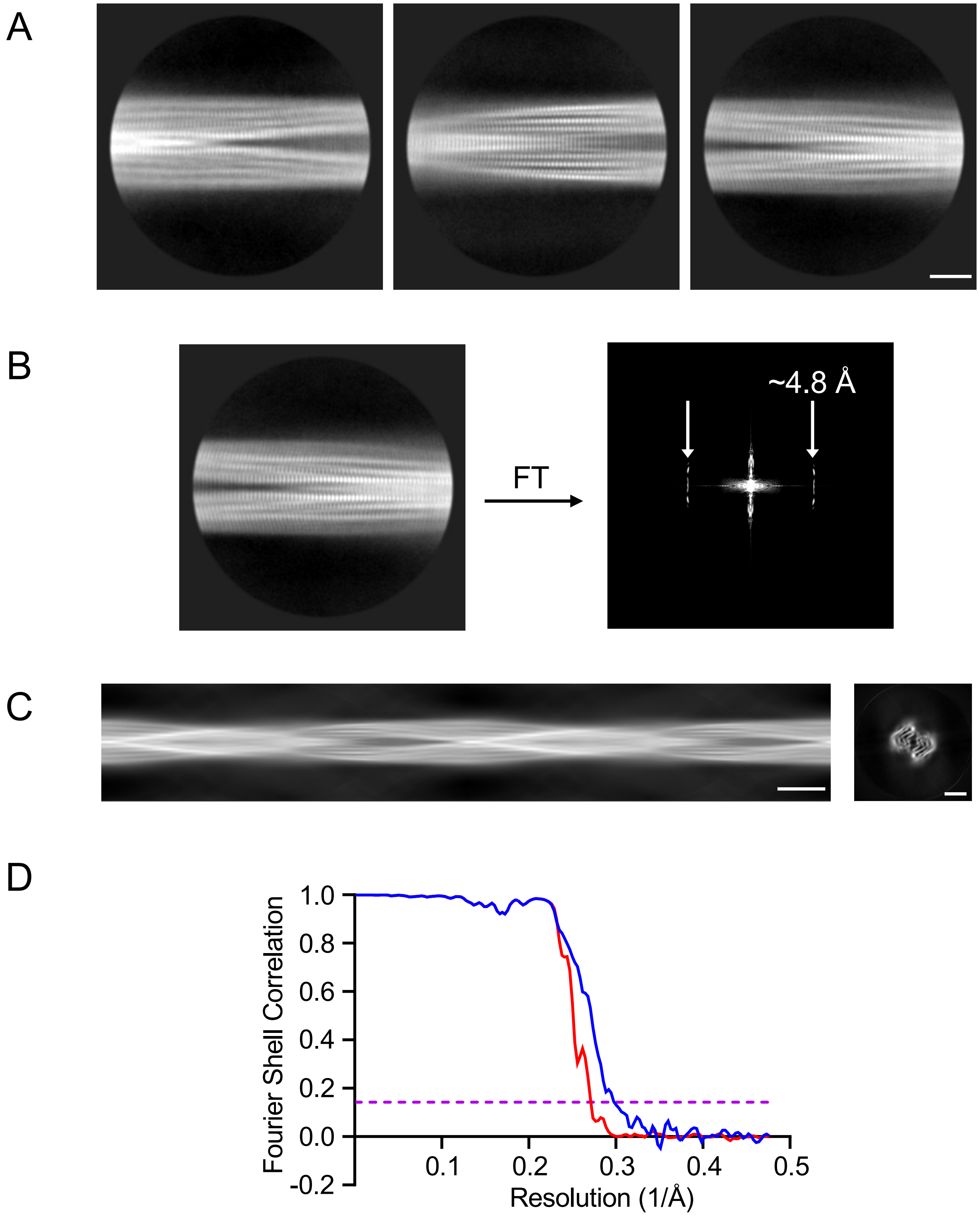


**Supplementary figure 4. 2D classes, cross-section and evaluation of the resolution of the cryo-EM high-resolution map of αSyn amyloid fibrils in complex with WT DNAJB1.**

A) 2D classes of αSyn amyloid fibrils in complex with WT DNAJB1. The DNAJB1 density was masked out to focus on fibril structure for this analysis. Scale bar, 50 Å.

B) A 2D class and its corresponding FT, showing the 4.8 Å repeat.

C) Side view of the aligned 2D classes and the calculated cross-section. Scale bars, side view 100 Å, cross section 50 Å.

D) Fourier shell correlation (FSC) curves of two independently refined half-maps with phase randomisation (red curve) and of the final cryo-EM reconstruction (blue curve). The dashed purple line indicates FSC = 0.143.

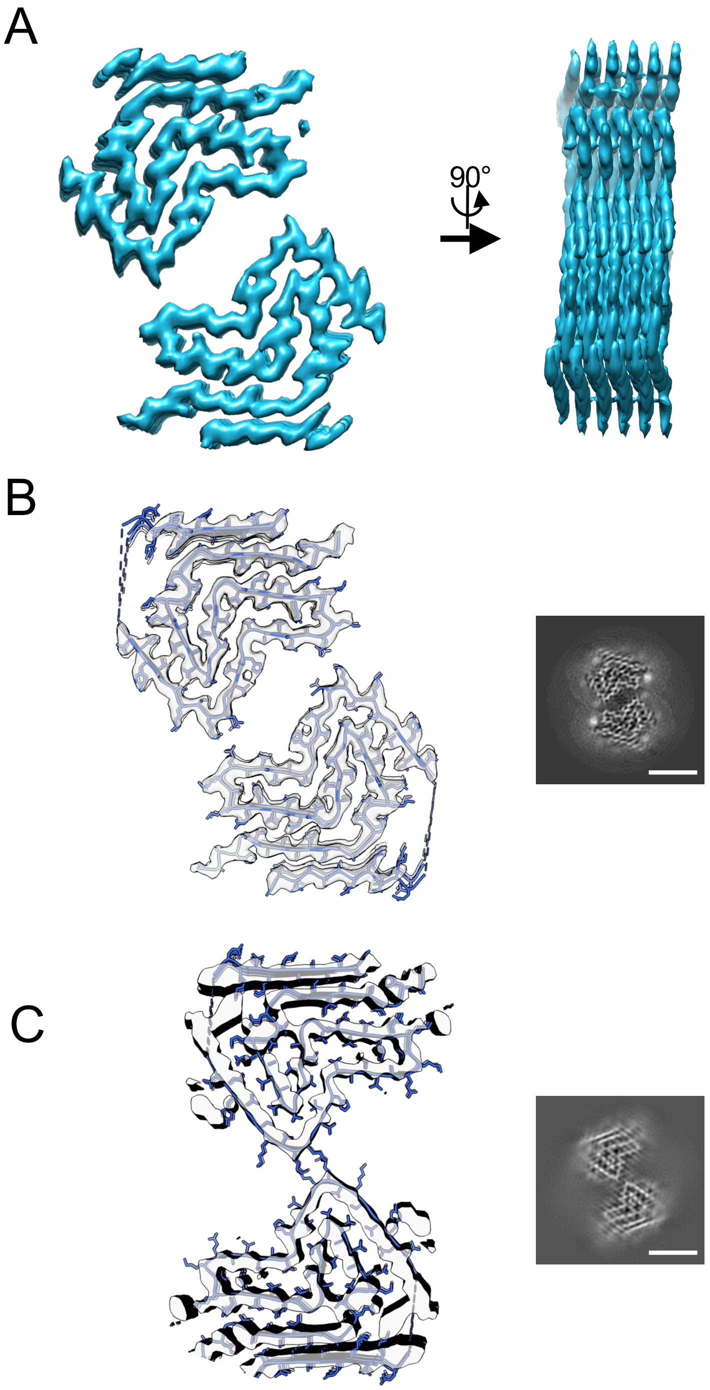


**Supplementary figure 5. Structure of αSyn amyloid fibril from the WT DNAJB1 dataset.**

A) Cryo-EM map (cross-section and side view) of the αSyn amyloid fibril polymorph 1 reconstruction.

B) Cryo-EM map of a single protofilament with the refined atomic model and the projected density slice of a single repeat. Scale bar, 50 Å.

C) Cryo-EM map of polymorph 2 with the rigidly docked atomic structure PDB:6SST (each protofilament docked independently). Scale bar, 50 Å.

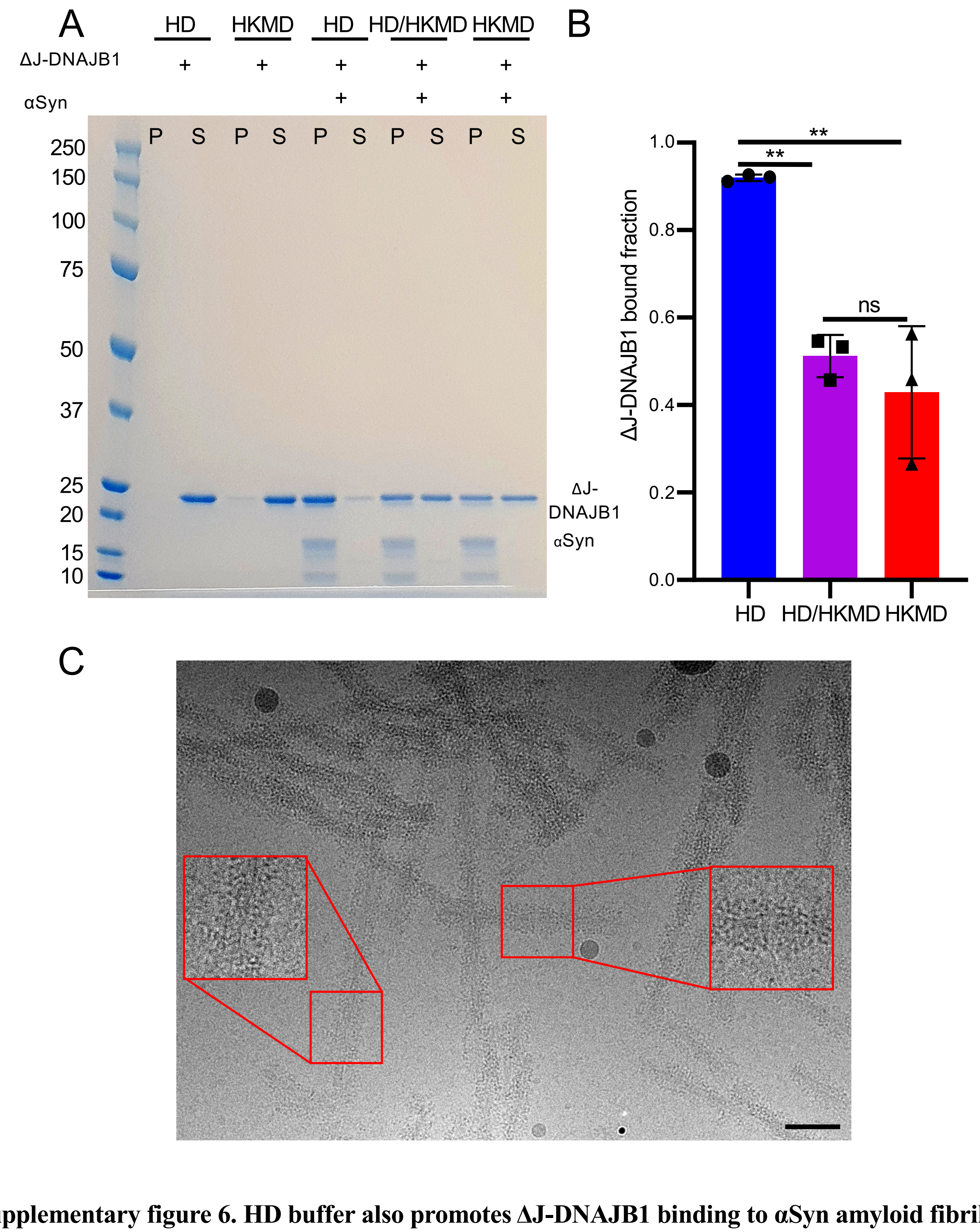


**Supplementary figure 6. HD buffer also promotes ΔJ-DNAJB1 binding to αSyn amyloid fibrils.**

A) Binding assay showing the reversibility of the binding when the salts are added back. The proteins were incubated twice in HD buffer (HD condition), twice in HKMD buffer (HKMD condition) or once in HD buffer and once in HKMD buffer (HD/HKMD buffer).

B) Histogram of ΔJ-DNAJB1 bound fraction in each condition shown in (A) (N = 3 independent experiments). A Shapiro-Wilk test was performed to check the normality of the data, followed by a one-way ANOVA with Tukey's multiple comparisons test (P = 0.0038 between HD and HD/HKMD conditions, P = 0.0015 between HD and HKMD conditions).

C) Micrograph showing αSyn amyloid fibrils decorated by ΔJ-DNAJB1 in HD buffer. Scale bar, 500 Å.

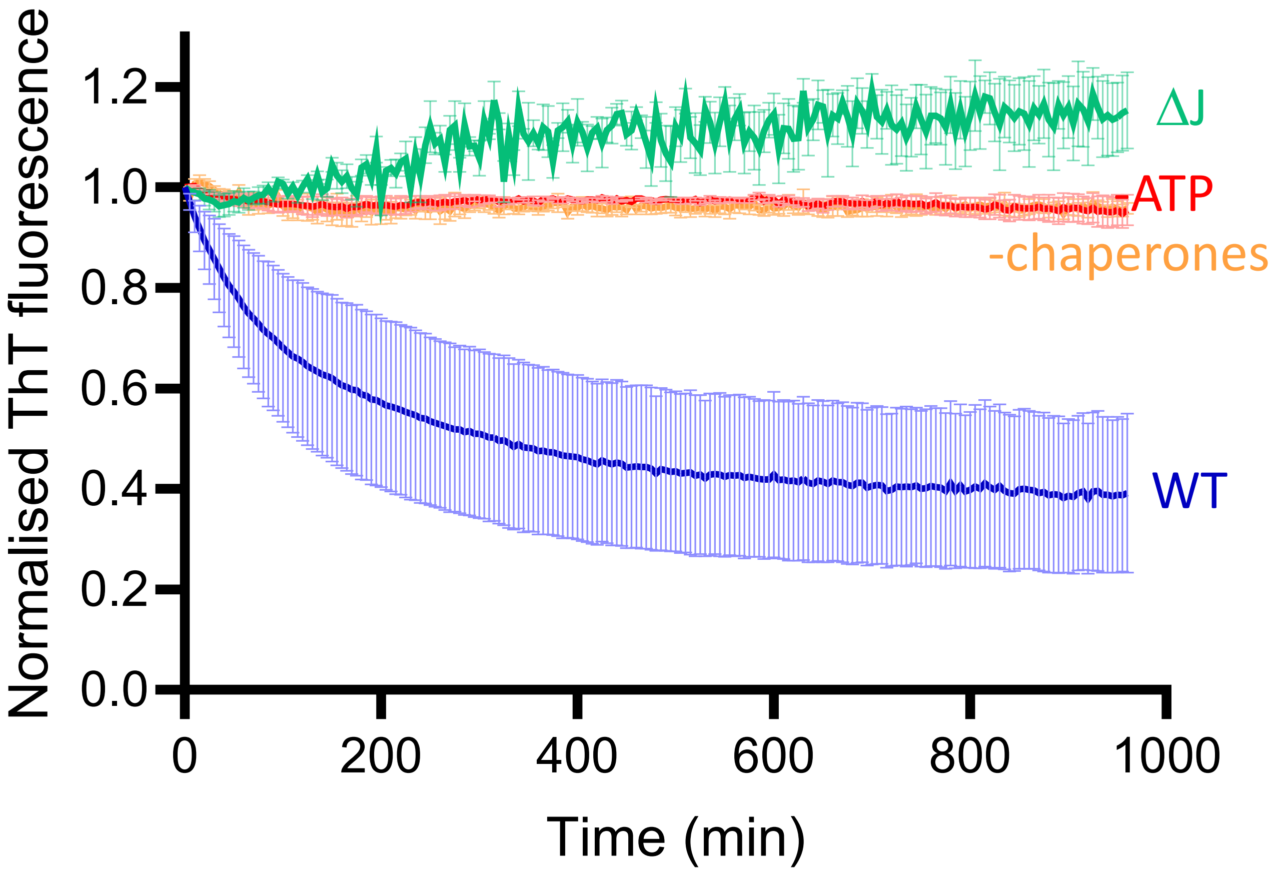


**Supplementary figure 7. DNAJB1 with the J domain deleted is completely inactive in disaggregation**. The disassembly of a-synuclein fibrils is monitored by the loss of thioflavin T fluorescence. Controls in which either the chaperones or the ATP is omitted are also inactive. Mean normalised ThT fluorescence with SEM is shown.

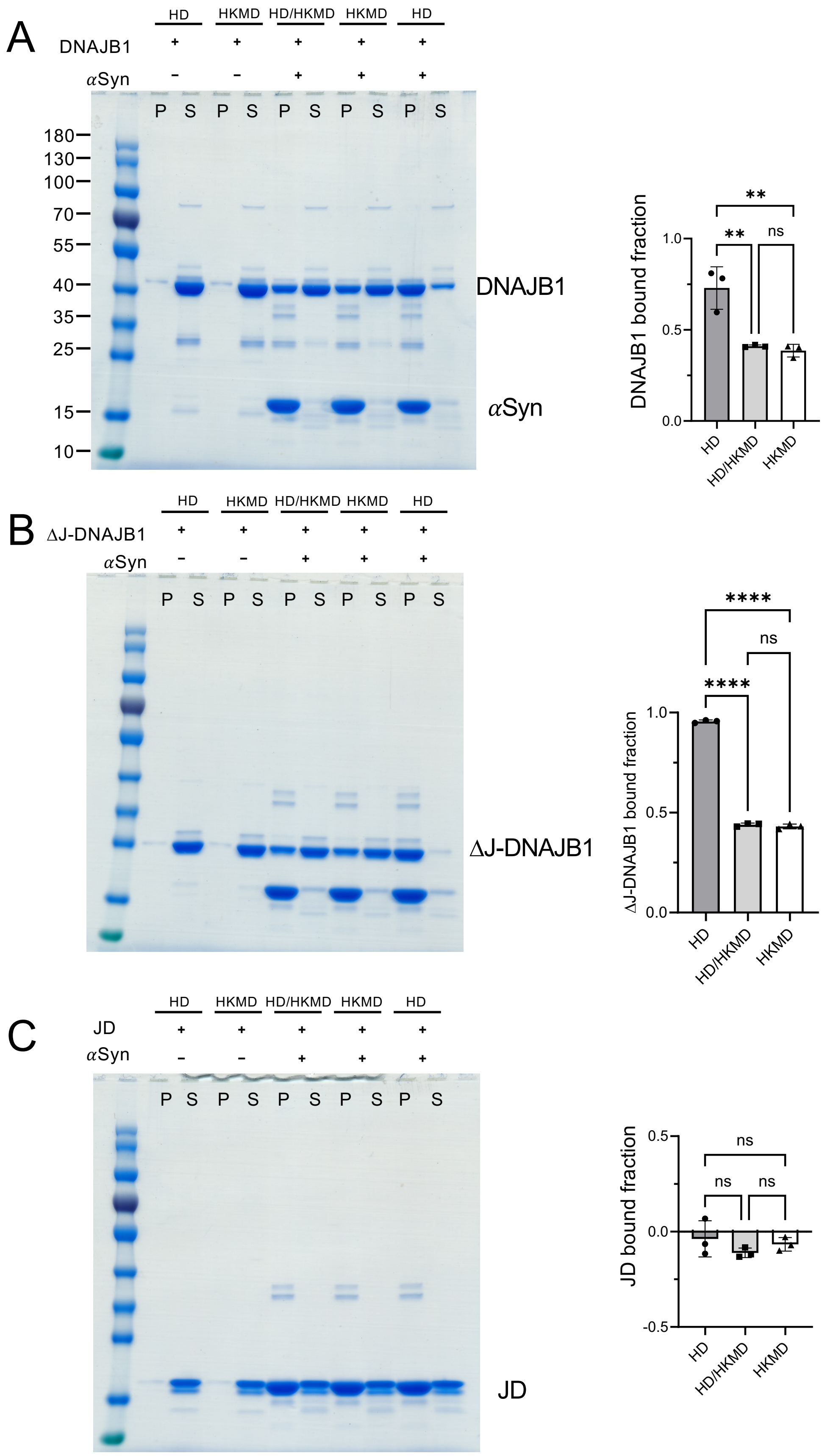


**Supplementary figure 8. Binding control for the isolated J-domain alone**.

The full length DNAJB1 (A) and ΔJ-DNAJB1 (B) binding assays were repeated and compared to the binding of the isolated J-domain (C). The bound fraction was estimated by measuring the unbound and subtracting from the total control band in the absence of αSyn because of the overlap between J-domain and αSyn bands in the pellet fraction. Histograms are shown for the averages of 3 experiments with the standard deviations. A Shapiro-Wilk test was performed to check the normality of the data followed by a one-way ANOVA with Tukey's multiple comparisons test. In (A), P = 0.0034 between HD and HD/HKMD and P = 0.0023 between HD and HKMD. In (B), P is smaller than 0.0001.

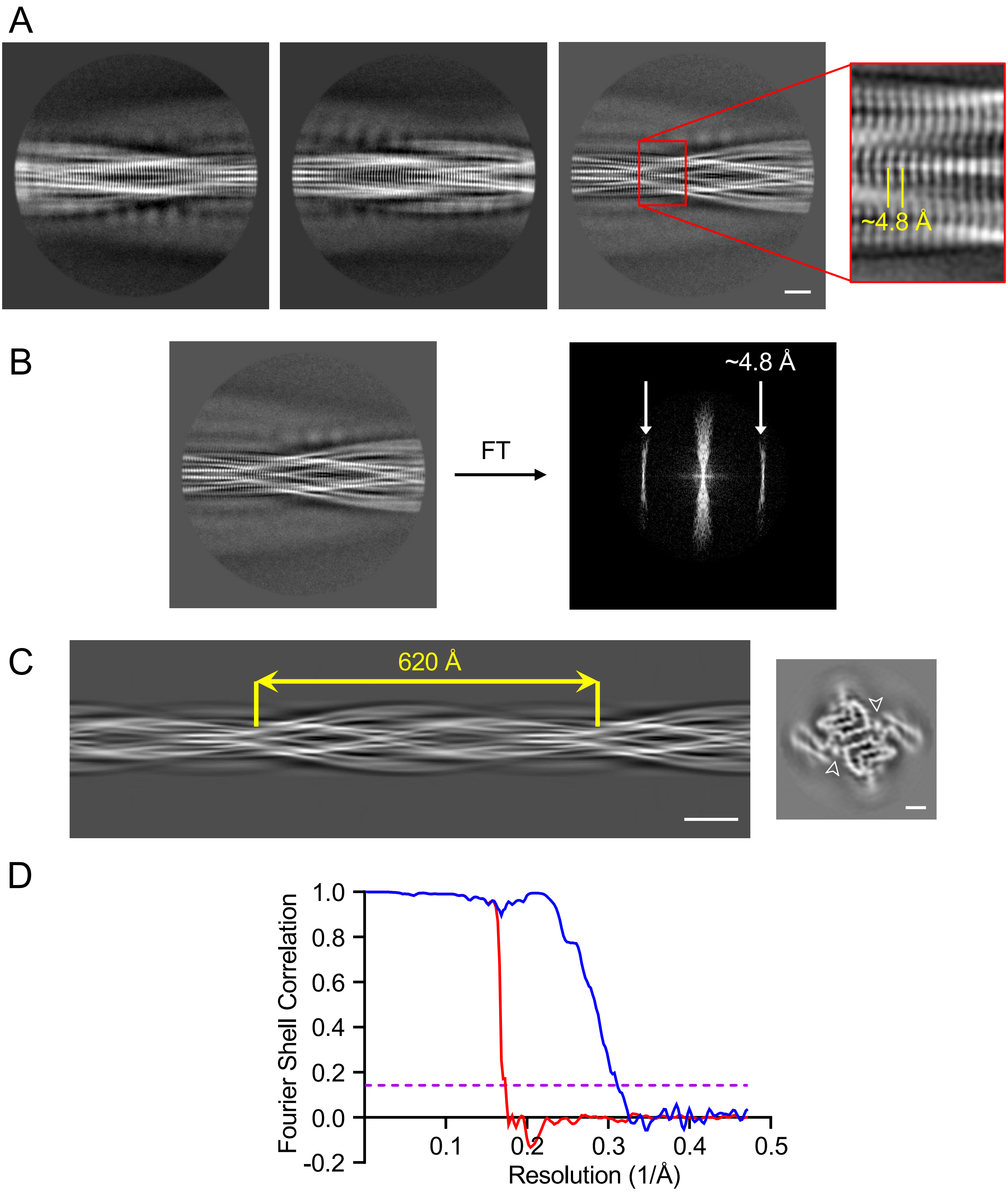


**Supplementary figure 9. 2D classes, cross-section and resolution plot of the cryo-EM map of αSyn amyloid fibrils in complex with ΔJ-DNAJB1.**

A) 2D classes of αSyn amyloid fibrils in complex with ΔJ-DNAJB1. A mask was applied to exclude ΔJ-DNAJB1 signal in the alignment. Scale bar, 50 Å.

B) A 2D class and its corresponding FT, showing a peak at around 4.8 Å.

C) Side view of the aligned 2D classes and the calculated cross-section. The crossover distance was estimated at 620 Å. Scale bars, 100 Å for the side view and 20 Å for the cross-section.

D) Fourier shell correlation (FSC) curves of two independently refined, masked half-maps using phase randomisation (red curve) and of the final cryo-EM reconstruction (blue curve). The dashed purple line indicates FSC = 0.143.

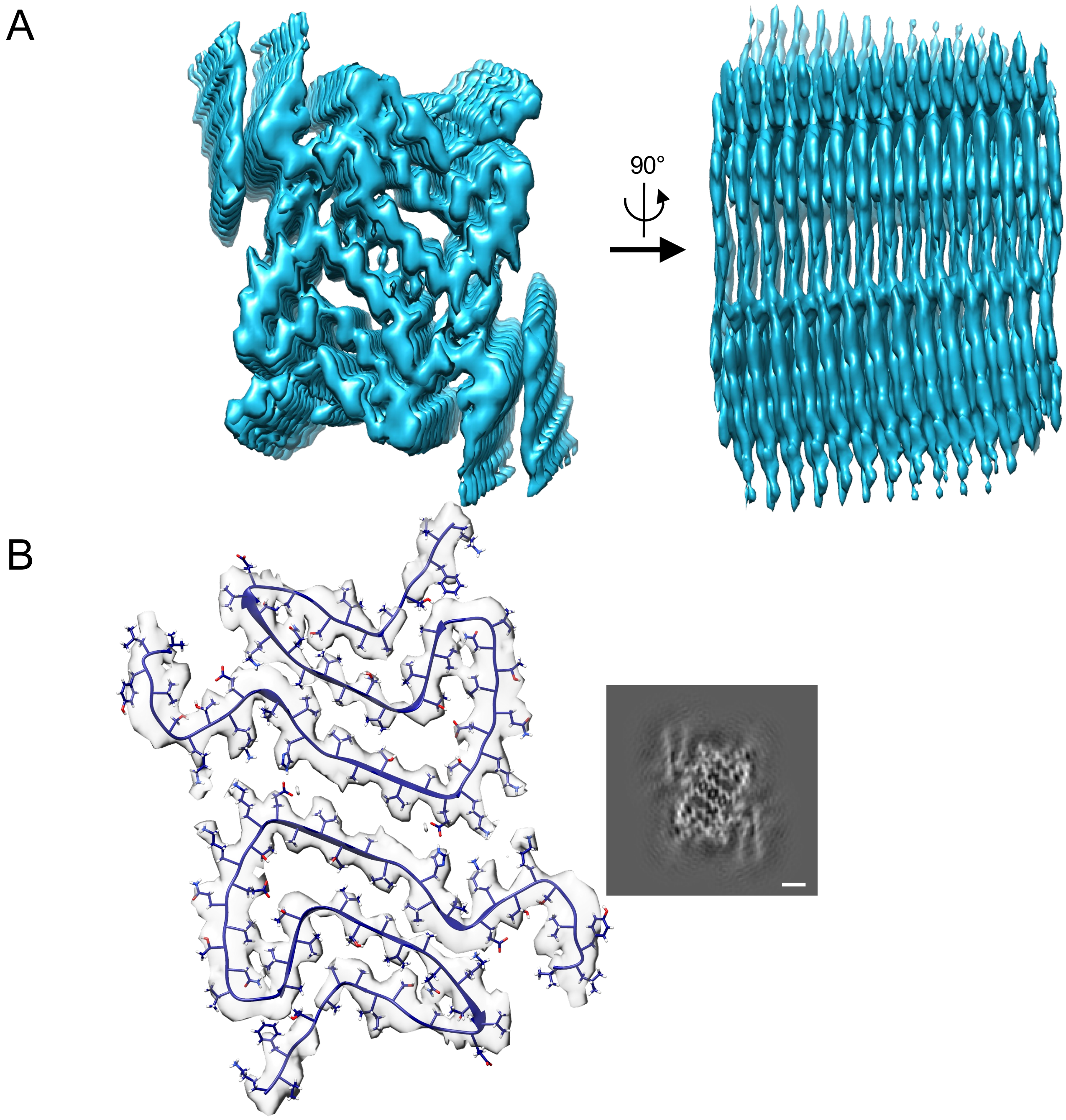


**Supplementary figure 10. Structure of αSyn amyloid fibril from the ΔJ-DNAJB1 dataset.**

A) Cryo-EM map (cross-section and side view) of the αSyn amyloid fibril.

B) Cryo-EM map of a single protofilament with the atomic model (PDB entry: 6OSJ) fitted into the density and the projected density slice of a single repeat. Scale bar, 20 Å.

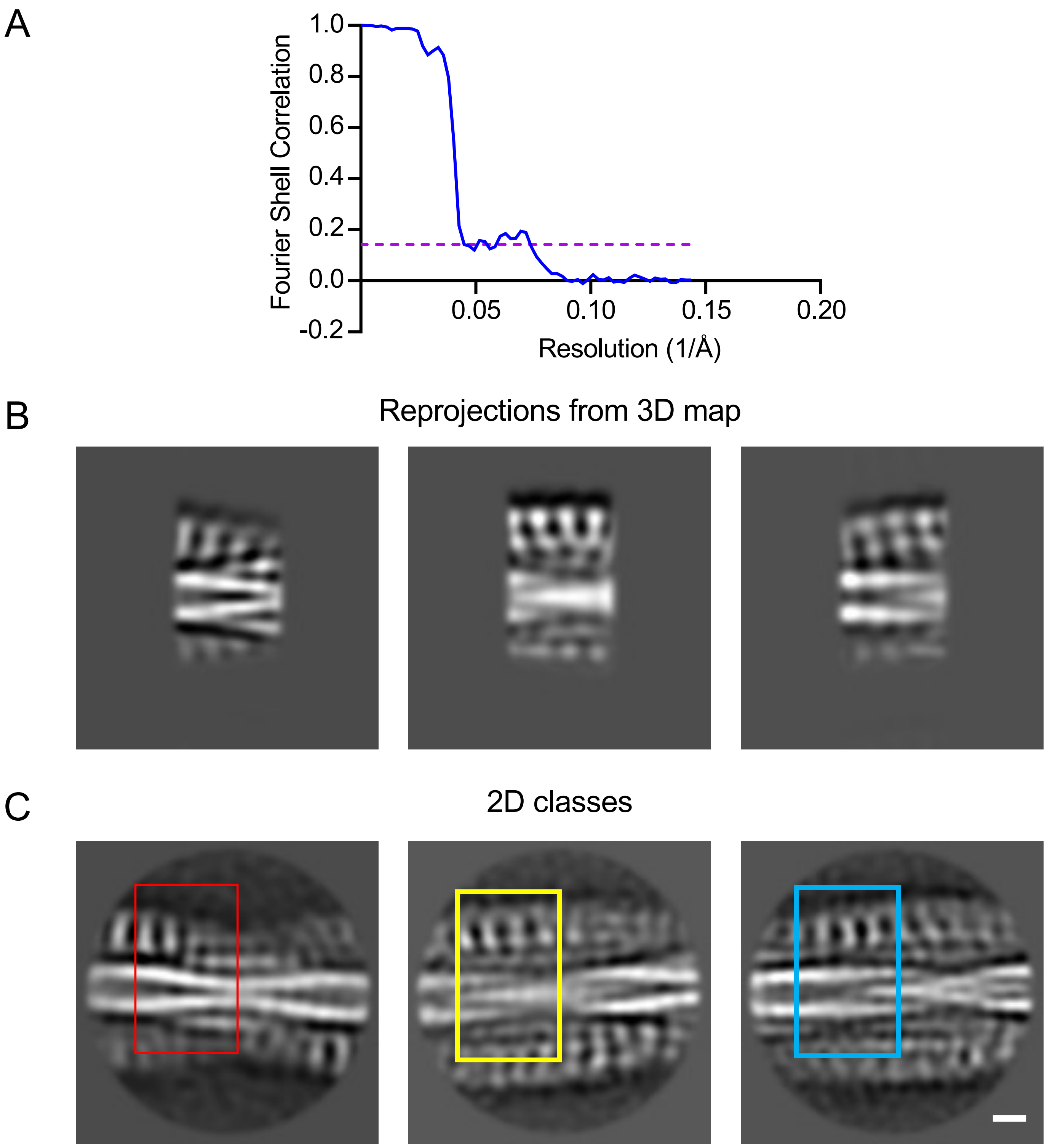


**Supplementary figure 11. 2D classes cross-section and resolution plot of ΔJ DNAJB1:αSyn fibril complex, 2D classes and reprojections from the map.**

A) FSC curve of the final cryo-EM reconstruction (blue curve). The dashed purple line indicates FSC = 0.143.

B) Side-view reprojections from the postprocessed map.

C) 2D class averages of the ΔJ DNAJB1:αSyn fibril complex. The 2D class averages resemble the reprojections. Scale bar, 50 Å.

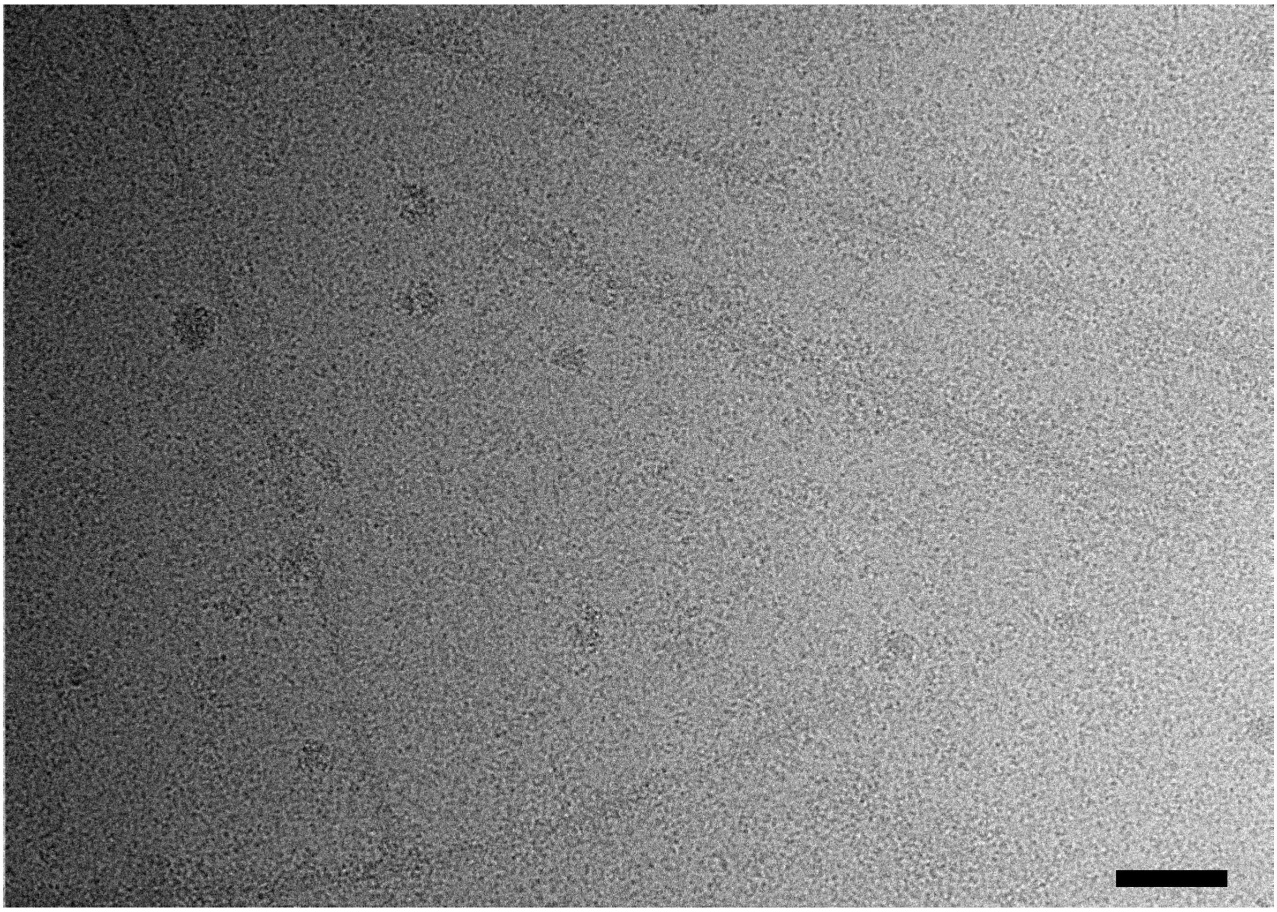

**Supplementary figure 12. Cryo-EM image of αSyn amyloid fibrils in the presence of DNAJB1, Hsc70, Apg2 and ATP in disaggregation buffer**. The poor contrast due to the high background of unbound chaperones and the lack of strict regularity of the complex precluded single particle analysis of this system. Scale bar, 500 Å.

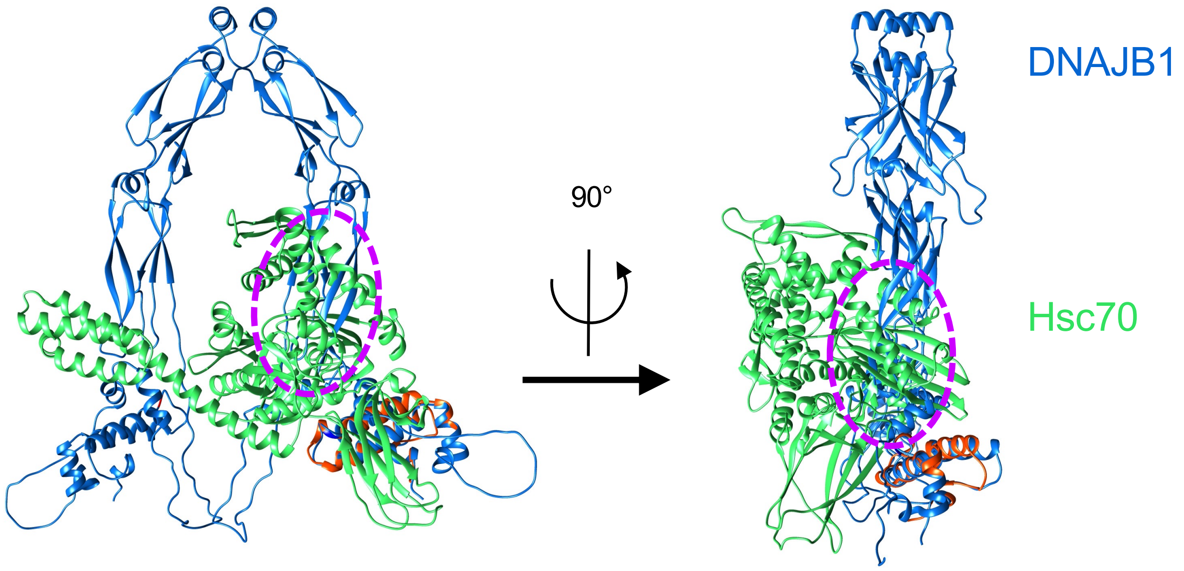


**Supplementary figure 13. Clash between Hsp70 (green) and DNAJB1 in auto inhibited form (blue).**

They are aligned via the J domain present in both structures (red), using a DnaK-J domain structure (PDB 5nro) and the DNAJB1 fit from Figure 2. With the J domain close to the C terminal domain, the ATPase domain of Hsc70 totally overlaps with the C terminal domain (magenta dashed outlines).
